# Development and validation of a high resolving power absolute quantitative per‐ and polyfluoroalkyl substances method incorporating Skyline data processing

**DOI:** 10.1002/rcm.9295

**Published:** 2022-03-25

**Authors:** Jeffrey R. Enders, Rebecca A. Weed, Emily H. Griffith, David C. Muddiman

**Affiliations:** ^1^ Molecular Education, Technology and Research Innovate Center (METRIC) North Carolina State University Raleigh North Carolina; ^2^ Department of Biological Sciences North Carolina State University Raleigh North Carolina; ^3^ Department of Statistics, College of Sciences North Carolina State University Raleigh North Carolina; ^4^ Department of Chemistry North Carolina State University Raleigh North Carolina

## Abstract

**Rationale:**

The ability to perform absolute quantitation and non‐targeted analysis on a single mass spectrometry instrument would be advantageous to many researchers studying per‐ and polyfluoroalkyl substances (PFAS). High‐resolution accurate mass (HRAM) instrumentation (typically deployed for non‐targeted work) carries several advantages over traditional triple quadrupole workflows when performing absolute quantitation. Processing this data using a vendor‐neutral software would promote collaboration for these environmental studies.

**Methods:**

LC‐MS (Orbitrap Exploris 240) was used for absolute quantitation of 45 PFAS using precursor (MS1) peak areas for quantitation, whereas isotope pattern matching and fragmentation (MS2) pattern matching were used for qualitative identification. In addition, a fluorinated chromatographic column achieved superior separation compared to the typical C18 columns typically used in PFAS analyses. This method was validated across eight different chemical classes using recommended guidelines found in EPA Method 537.1 and Skyline data processing software.

**Results:**

The validated limits of all 45 compounds, as well as metrics or accuracy and reproducibility, are reported. Most compounds achieved limits of quantitation in the range of 2‐50 ng/L. Four newly released Chemours‐specific compounds (PEPA, PFO3OA, PFO4DA, and PFO5DoA) were also validated. Aspects of data analysis specific to high resolving power absolute quantitation are reviewed as are the details of processing these data via Skyline.

**Conclusions:**

This method shows the feasibility of performing reproducible absolute quantitation of PFAS on an HRAM platform and does so using an open‐source vendor‐neutral data processing software to facilitate sharing of data across labs and institutions.

## 1 INTRODUCTION

Per‐ and polyfluoroalkyl substances (PFAS) have been produced in the United States since the 1940s and consist of long carbon chains with fluorine atoms in place of hydrogen atoms and often a hydrophilic functional group. These two characteristics together bestow a unique solubility property on these compounds that makes them both hydrophobic and lipophilic (hence their value to consumer product applications). Unfortunately, these same advantageous properties are the cause of much controversy; because PFAS are both water and fat repellent, they are resistant to almost all forms of degradation and are therefore highly persistent in the environment and subsequently in biological organisms present in that environment.

Liquid chromatography‐mass spectrometry (LC‐MS) is a natural choice for analyzing this class of compounds. The use of aqueous and organic mobile phases to enact a separation of analytes across a reversed‐phase (hydrophobic) column based on analyte hydrophobicity has been shown to handle the unusual solubility of these chemicals well.^1^ Negative polarity electrospray ionization (ESI) is used to great effect for almost all PFAS currently being analyzed. There have been numerous papers detailing detection and quantitation of PFAS from a number of different matrices, including water, blood, and tissue using reversed‐phase LC‐MS.^2^, ^3^, ^4^, ^5^, ^6^ Many of these workflows use solid‐phase extraction (SPE) to isolate and selectively remove matrix interferences and occasionally to concentrate analytes of interest in low‐concentration samples (such as drinking water). However, these extraction steps can be monetarily and temporally costly. One method of reaching low‐level limits (in relatively clean matrices) without the need of SPE concentration steps is to increase the volume of sample that is injected onto the LC column. With injection volumes as high as 900 μL, various PFAS compounds have successfully been quantitated below 1 ng/L.^7^ When using this strategy, careful management of chromatographic conditions is necessary to avoid unwanted effects of such a large bolus of sample volume being injected into the flow path. However, employing methods in this manner can allow for quantitation of PFAS at levels relevant for drinking water analysis (i.e., in the low to mid parts per trillion or ng/L range) without the need for pre‐concentration and associated sample handling.^8^

With regard to mass analysis of PFAS, two main workflow trends predominate: MS/MS analysis via a triple quadrupole instrument for quantitation^9^ and high resolution accurate mass (HRAM) instrumentation (e.g., orbitrap or Q‐TOF) for the identification of unknowns or emerging compounds^10^, ^11^ (i.e., those without authentic standards). Triple quadrupole instrumentation is generally regarded as being the most sensitive form of small molecule MS/MS analysis, whereas HRAM instrumentation provides superior qualitative metrics such as exact mass, full precursor scan MS1 data, and full product ion scan MS2 data—with the possibility of fragment data for every eluted species if using techniques such as data‐independent acquisition (DIA), thus providing the opportunity for future re‐evaluation in the event that new or emerging PFAS are later suspected of being present.

Quantitative MS/MS analysis using a triple quadrupole instrument, often referred to as multiple reaction monitoring (MRM), requires lengthy method development and extensive validation testing to ensure data quality. Analyte transitions are easy to find in application notes from instrument vendors and in the literature, but oftentimes these transitions are optimized for a specific instrument model and do not produce optimal performance if programmed into another instrument type, let alone an instrument from a different vendor. In addition, frequent changes of the method to incorporate or remove new or emerging compounds can become challenging and may necessitate further validation of the method to ensure opportunities for interference have not been introduced.

With this instrument workflow, quantitation is performed on the product ion and therefore the abundance of this product ion signal governs the method's sensitivity. In addition, because a quadrupole is inherently not very specific (typically providing only unit mass resolving power), monitoring for products that are specific to an analyte is crucial, and further, analyzing at least two transitions per analyte and the subsequent ratio between the signal from these two transitions (i.e., the ion ratio) can be quite important in providing high‐quality data. This is especially important in the case of PFAS, where there are many nominal mass isobars that produce nominal mass isobaric fragments. For these reasons, analyte transitions and associated transition‐specific settings must be fully optimized for all analytes, thus necessitating a lengthy method development process.

Alternatively, high resolving power mass analysis (e.g., using TOF or orbitrap technology) is equipped to provide full range mass data for both precursor and product scans. This negates much of the method development requirements inherent in triple quadrupole use because of the fact that full scan MS1 data provide precursor ion exact masses that can be used for quantitation (in the absence of coeluting exact mass isobars). If desired, quantitation can also be carried out on the MS2 fragment ion chromatographic traces, in a manner similar to triple quadrupole workflows.^12^ Furthermore, once selected for fragmentation, the product ion scans provide information on the entire *m/z* range, thus augmenting the qualitative data on the analytes and helping to reduce false positives. With HRAM instrumentation and a properly developed method, it is possible, with one scan event, to measure the exact mass of the precursor, the precursor isotopic pattern, the retention time, and exact mass MS2 data for all product ions generated, thus providing an extremely robust identification and quantitative value when compared to known standards. Measuring for analytes in this way using HRAM instrumentation provides a number of key advantages over low resolving power (e.g., triple quadrupole) instrumentation. The use of exact mass precursors for quantitation avoids the pitfalls of interference‐related nominal mass resolving power issues—only co‐eluting exact mass isobars would potentially still cause an issue. This theoretically grants the use of more crowded chromatography and therefore a shorter method (as long as suppression effects are properly monitored) as exact mass can be used to discriminate the analytes of interest. In addition, the ability of the HRAM instrument to collect an entire mass range for each product ion scan provides additional qualitative metrics with which to judge the identification of the analyte of interest. In the absence of any interfering species, the identity and relative abundance of product ions for a given molecule are consistent for a given instrument and group of voltage (source and collision) and gas settings across all concentration ranges of that molecule up to the point of instrument detector saturation. As such, monitoring for these fragments and their relative ratios to one another is a very useful means of controlling for data quality and is already used quite heavily in proteomic quantitative workflows.^13^

Environmental PFAS analysis is a highly collaborative endeavor especially with regard to the analytical aspects of this field of research. The use of mass spectrometer instrument vendor‐specific software to process, display, and share data greatly limits peer review of this part of the workflow. Taking advantage of a vendor‐neutral data processing platform, such as Skyline,^14^ which already provides resources for data set sharing, should greatly advance PFAS method development and data processing strategies.

To this end, this work presents a HRAM method for 45 PFAS analytes and 23 internal standards developed on an orbitrap platform that utilizes exact mass precursor ion signal absolute quantitation and product ion signal qualitative controls. Data processing is performed using the open‐source vendor‐neutral Skyline software.

## 2 MATERIALS AND METHODS

### 2.1 Standards

All experiments were performed on an Orbitrap Exploris 240 (Thermo Scientific, Bremen, Germany) incorporating a Thermo Scientific Vanquish LC system (Germering, Germany). Two unlabeled PFAS mixtures were acquired from Cambridge Isotope Laboratories (Tewksbury, MA, USA). Mix 1 (2 μg/mL each) contained 1H,1H,2H,2H‐perfluorohexane sulfonate, Na salt (4:2 FTS), 1H,1H,2H,2H‐perfluorooctane sulfonate, Na salt (6:2 FTS), 1H,1H,2H,2H‐perfluorodecane sulfonate, Na salt (8:2 FTS), 1H,1H,2H,2H‐perfluorododecane sulfonate, Na salt (10:2 FTS), *N*‐ethylperfluoro‐octanesulfonamidoacetic acid (mixed isomers) (NEtFOSAA), *N*‐methylperfluoro‐octanesulfonamidoacetic acid (mixed isomers) (NMeFOSAA), perfluorooctanesulfonamide (PFOSA), *N*‐methylperfluorooctanesulfonamide (MeFOSA), perfluorobutane sulfonate, K salt (PFBS), perfluorohexane sulfonate (mixed isomers), K salt (PFHxS), perfluorooctane sulfonate (mixed isomers) (PFOS), perfluorobutyric acid (PFBA), perfluoropentanoic acid, Na salt (PFPeA), perfluorohexanoic acid, Na salt (PFHxA), perfluoroheptanoic acid (PFHpA), perfluorooctanoic acid (PFOA), perfluorononanoic acid (PFNA), perfluorodecanoic acid, Na salt (PFDA), perfluoroundecanoic acid, Na salt (PFUdA), perfluorododecanoic acid, Na salt (PFDoA), perfluorotetradecanoic acid (PFTeDA), and tetrafluoro‐2‐(heptafluoropropoxy)propanoic acid (HFPO‐DA [Gen‐X]). Mix 2 (1 μg/mL each) contained perfluorobutane sulfonamide (FBSA), perfluorohexane sulfonamide (FHxSA), perfluorohexadecanoic acid (PFHxDA), perfluorooctadecanoic acid (PFODA), perfluoropentane sulfonate, Na salt (PFPeS), perfluoroheptane sulfonate, Na salt (PFHpS), perfluorononane sulfonate (linear), Na salt (PFNS), perfluorodecane sulfonate, Na salt (PFDS), perfluorotridecanoic acid (PFTrDA), 7:3 fluorotelomer carboxylic acid, FHpPA, 3‐perfluoroheptyl propanoic acid(7:3) (7:3 FT[C]A), *N*‐(3‐dimethylaminopropan‐1‐yl)perfluoro‐1‐hexane‐sulfonamide (*N*‐AP‐FHxSA), *N*‐(carboxymethyl)‐*N*,*N*‐dimethyl‐*N*‐[3‐(1H,1H,2H,2H‐perfluoro‐1‐octanesulfonamido)propan‐1‐yl]ammonium (*N*‐CMAmP‐6:2FOSA [6:2 FTAB]), *N*‐[3‐(perfluoro‐1‐hexanesulfonamido)propan‐1‐yl]‐*N*,*N*,*N*‐trimethylammonium (*N*‐TAmP‐FHxSA), sodium dodecafluoro‐3H‐4,8‐dioxanonanoate (NaDONA), ethanesulfonic acid, 2‐[1‐[difluoro[(1,2,2‐trifluoroethenyl)oxy]methyl]‐1,2,2,2‐tetrafluoroethoxy]‐1,1,2,2‐tetrafluoro, perfluoro‐3,6‐dioxa‐4‐methyl‐7‐octenesulfonic acid (Nafion by product 1, also known as PS Acid), ethanesulfonic acid, 2‐[1‐[difluoro(1,2,2,2‐tetrafluoroethoxy)methyl]‐1,2,2,2‐tetrafluoroethoxy]‐1,1,2,2‐tetrafluoro‐polymer with 1,1,2,2‐tetrafluoroethene, 7H‐perfluoro‐4‐methyl‐3,6‐dioxaoctanesulfonic acid (Nafion by product 2), 11‐chloroeicosafluoro‐3‐oxaundecane‐1‐sulfonic acid (F53B Minor [11Cl‐PF3OUdS]), 9‐chlorohexadecafluoro‐3‐oxanone‐1‐sulfonic acid (F53B Major [9Cl‐PF3ONS]), and perfluoro‐2‐methoxyacetic acid (PFMOAA). 2,3,3,3‐Tetrafluoro‐2‐(1,1,2,2,2‐pentafluoroethoxy)propanoate (PEPA), sodium nonafluoro‐2,4,6‐trioxaoctan‐8‐oate (PFO3OA), sodium undecafluoro‐2,4,6,8‐tetraoxadecan‐10‐oate (PFO4DA), and sodium tridecafluoro‐2,4,6,8,10‐pentaoxadodecan‐12‐oate (PFO5DoA) were acquired from Fluoryx Labs (Carson City, NV, USA).

A stable isotope–labeled internal standard mixture (1 μg/mL) was also prepared by Cambridge Isotope Laboratories and contained the following: 1H,1H,2H,2H‐perfluorohexane sulfonate, Na salt (^13^C_2_/D_4_) (^13^C_2_/D_4_‐4:2 FTS), 1H,1H,2H,2H‐perfluorooctane sulfonate, Na salt (^13^C_2_/D_4_) (^13^C_2_/D_4_‐6:2 FTS), 1H,1H,2H,2H‐perfluorodecane sulfonate, Na salt (^13^C_2_/D_4_) (^13^C_2_/D_4_–8:2 FTS), 1H,1H,2H,2H‐perfluorododecane sulfonate, Na salt (^13^C_2_/D_4_) (^13^C_2_/D_4_–10:2 FTS), *N*‐ethylperfluorooctanesulfonamidoacetic acid (D_5_) (D_5_‐NEtFOSAA), *N*‐methylperfluorooctanesulfonamidoacetic acid (D_3_) (D_3_‐NMeFOSAA), perfluoro‐1‐[^13^C_8_]octanesulfonamide (^13^C_8_‐PFOSA), *N*‐methylperfluorooctanesulfonamide (D_3_) (D_3_‐MeFOSA), perfluorobutane sulfonate, K salt (^13^C_4_) (^13^C_4_‐PFBS), perfluorohexane sulfonate, K salt (^13^C_6_) (^13^C_6_‐PFHxS), perfluorooctane sulfonate, Na salt (^13^C_8_) (^13^C_8_‐PFOS), perfluorobutyric acid, Na salt (^13^C_3_) (^13^C_3_‐PFBA), perfluoropentanoic acid, Na salt (^13^C_5_) (^13^C_5_‐PFPeA), perfluorohexanoic acid, Na salt (^13^C_6_) (^13^C_6_‐PFHxA), perfluoroheptanoic acid, Na salt (^13^C_7_) (^13^C_7_‐PFHpA), perfluorooctanoic acid (^13^C_8_) (^13^C_8_‐PFOA), perfluorononanoic acid (^13^C_9_) (^13^C_9_‐PFNA), perfluorodecanoic acid (^13^C_9_) (^13^C_9_‐PFDA), perfluoroundecanoic acid, Na salt (^13^C_9_) (^13^C_9_‐PFUdA), perfluorododecanoic acid, Na salt (^13^C_12_) (^13^C_12_‐PFDoA), perfluoro‐*n*‐[1,2‐^13^C_2_]tetradecanoic acid (^13^C_2_‐PFTeDA), perfluoro‐*n*‐[1,2‐^13^C_2_]hexadecanoic acid (^13^C_2_‐PFHxDA), and tetrafluoro (heptafluoropropoxy)[^13^C_3_]propanoic acid (^13^C_3_‐HFPO‐DA [Gen‐X]).

### 2.2 Sample preparation

The internal standard was prepared at 10 000 ng/L by diluting the stock in 50:50 MeOH:water with 0.1% formic acid using volumetric glassware. Calibrators were prepared by combining the appropriate volumes of mix 1 and mix 2 and diluting to mark with 50:50 MeOH:water with 0.1% formic acid using volumetric glassware. Internal standard and calibrators were mixed in the ratio of 10:1 (e.g., 900 μL of calibrator mixed with 100 μL of IS) to produce a final sample composition of 50% MeOH. All solutions were stored in polypropylene (PP) conical tubes. Maintaining a minimum organic composition of 50% was necessary to ensure solubility of the hydrophobic compounds to the walls of these PP tubes and LC vials.

### 2.3 LC‐MS method

A reversed‐phase LC setup consisting of a Thermo Scientific Vanquish LC system (Germering, Germany) was used. Aqueous (solvent A: water with 5% ACN and 0.1% formic acid) and organic (solvent B: ACN with 5% water and 0.1% formic acid) solvents were run at 500 μL/min using the following gradient: 0 min: 1% B, 2 min 1% B, 13 min: 70% B, 13.01 min: 99% B, 17 min: 99% B, 17.01 min: 1% B, 20 min 1% B. A Phenomenex Kinetex 2.6 μm particle, F5 100 Å, 100 × 2.1 mm analytical column (part number 00D‐4723‐AN) was used for separation. A Thermo Scientific 1.9 μm particle Hypersil GOLD column, 50 × 3.0 mm (25002‐053030) was placed in the flow path after the binary pumps but before the autosampler to delay any potential PFAS present in the LC solvents and thus prevent their integration into the sample peak area. The autosampler was operated at 10°C, whereas the column compartment was set to 45°C. A 100 μL sample loop was used to accommodate a 100 μL injection volume. The system was also upgraded to be “PFAS ready” by Thermo Fisher Scientific (Bremen, Germany).

For mass analysis, an Orbitrap Exploris 240 (Thermo Fisher Scientific) was used. A negative mode full scan event at 60 000 resolving power at *m/z* = 200_FWHM_ was “triggered” for MS2 by detection of an exact mass as dictated by a list of analytes that was entered into a targeted mass filter. The MS2 scan was run at 15 000 resolving power at *m/z* = 200_FWHM_ with dynamic exclusion turned off. A second identical but positive mode event was run from minutes 9.5 to 11.5 to capture the zwitterions (*N*‐AP‐FHxSA, *N*‐CMAmP‐6:2FOSA, and *N*‐TAmP‐FHxSA). Optimized settings for the various analytes in this panel were often mutually exclusive (i.e., the most ideal settings for some analytes were the least ideal for other analytes). These differences often manifested according to analyte mass/chain length/hydrophobicity. As such, dynamic source settings were used. Negative spray voltage was (−) 1000 V from 0 to 11.6 min and (−) 1500 V from 11.6 to 20 min. From 0 to 11.6 min sheath gas flow/Aux gas flow/Sweep gas flow were set to 50/12/0.5, and from 11.6 to 20 min, these same settings were at 30/10/1. The ion transfer tube temperature was 240°C, and the vaporizer temperature was 300°C. In addition, two MS1 mass ranges were used. For the lower mass analytes, a range of 50‐600 Da was optimal; however, these settings rendered many high mass analytes undetectable. For this reason a second MS1 event that incorporated a mass range of 150‐1500 Da was used. Although this mass range also captured the lower mass analytes, their sensitivity was drastically reduced under these conditions.

### 2.4 Skyline data processing

Skyline Daily, version 21.1.1.298 was used for data processing. Thermo data (.RAW) were imported into Skyline without any need for manipulation or conversion. PFAS target lists were generated using the “Edit → insert → transition list” function. Analyte precursor masses were entered (based on molecular formulas) as well as product ion masses that were observed upon infusion of pure standards (after collision energy optimization) on the instrument.

### 2.5 Method validation

The method was validated in neat solvents (i.e., water and methanol), roughly in accordance with guidance detailed in EPA Method 537.1. Minimum reporting limit (MRL) and detection limit (DL) were determined as is detailed in this same method. Briefly, 5‐10 replicates of the calibration curve were prepared and run. By treating one of the replicates as the calibration curve and the others as unknowns, a large amount of replicate calibration curve data is produced. The half range for the prediction interval of results (HR_PIR_) was calculated as follows:

$${\mathrm{HR}}_{\mathrm{PIR}}=s\times t_{\mathrm{df},\;1-\frac{.01}{2}}\times \left(1+\frac{1}{N}\right),$$

where *s* is the standard deviation, $t_{\mathrm{df},\;1-\frac{.01}{2}}$ is the two‐tailed 99% confidence interval student *t* value for the appropriate degrees of freedom, and *N* is the number of replicates used. Then, to determine the MRL, a calibration curve point's replicate data were required to meet the following criteria:

The upper PIR limit must be ≤150% recovery:

$$\frac{\text{Mean}+{\mathrm{HR}}_{\mathrm{PIR}}}{\text{Actual concentration}}\times 100\%\leq 150\%.$$

The lower PIR limit must be ≥50% recovery:

$$\frac{\text{Mean}-{\mathrm{HR}}_{\mathrm{PIR}}}{\text{Actual concentration}}\times 100\%\geq 50\%.$$

The DL was determined by subjecting the MRL concentration's data to the following calculation:

$$\mathrm{DL}=s\times t_{\mathrm{df},\;1-\frac{.01}{2}}.$$

The initial demonstration of precision (IDP) is the relative standard deviation of the replicate data at a mid‐level concentration (in this case 500 ng/L), whereas the initial demonstration of accuracy (IDA) was the accuracy of the replicate data compared to the prepared concentration at a mid‐level concentration (in this case 500 ng/L).

## 3 RESULTS AND DISCUSSION

### 3.1 Acquisition method development

**FIGURE 1:**
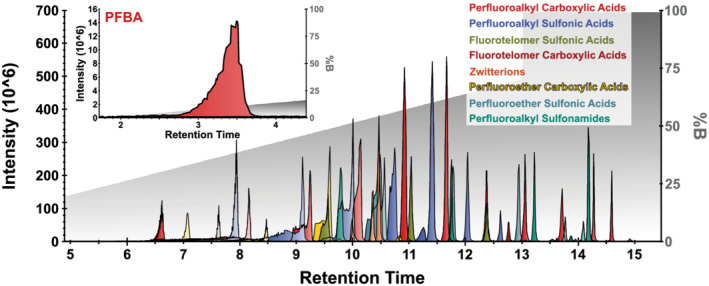
Extracted ion chromatograms for all 45 compounds. The gray shading in the background indicates the chromatographic gradient applied [Color figure can be viewed at wileyonlinelibrary.com]

Several column chemistries were explored; however, ultimately, the F5 fluorinated column from Phenomenex provided superior separation and retention under these gradient conditions and the following method restrictions (Figure 1). Various gradient elution profiles and starting percentages of mobile phase B were examined, though a low percentage of mobile phase B was considered important for cycling the hydrophobicity of the column to ensure elution of highly hydrophilic species. The C18 column appeared to have very high but somewhat non‐specific retention of PFAS compounds, whereas the F5 exhibited less retention (or retention more easily overcome by an organic mobile phase gradient) but a more specific retention profile when starting from a low percentage of mobile phase B. Using the F5 column in this manner allowed for a much shorter gradient length (temporally). This retention profile of the F5 column also seemed to allow it to handle the uncharacteristically large injection volume much more amenably.

Although mobile phase additives (e.g., ammonium acetate) were also explored, they did not appear to provide any improvement in peak shape or a desirable retention profile under these conditions and/or with this column.

All samples and standards were maintained at a 50% methanol composition to negate adsorption of the more hydrophobic PFAS to the walls of conical tubes and LC vials (both of which were polypropylene). Injecting 100 μL of a 50% methanol sample bolus presented numerous challenges for the more hydrophilic compounds (e.g., PFBA). These early eluting hydrophilic compounds will tend to produce very elongated and non‐Gaussian peak shapes as the high organic content bolus begins to mix with the low organic solvent gradient that surrounds it prior to it reaching the analytical column. As the bolus reaches the analytical column, there is a rapid transition of hydrophilic analytes between mobile and stationary phases, and some of the analytes may be retained very minimally (“breakthrough”). This can lead to a binomial distribution with minimally retained and more retained compounds separated slightly in retention time and can be dependent on many factors including column age. Although these peak shapes are not the traditional Gaussian shape, they remain reproducible and provide high quality quantitative values at the concentrations validated here. Later‐eluting hydrophobic analytes experience much less breakthrough and have much more regularly distributed peak shapes (Figure 1).

The Exploris 240 provides options for many scan functions to detect and quantitate the PFAS species. Many of the scan functions and associated settings were closely examined. For this method, priority was given to the compounds for which a standard curve was prepared, such that detection of species outside of this set was a secondary concern or not a concern because they could not be confidently quantitated. In addition, performing an MS1‐only scan followed by a secondary injection for investigation of fragmentation was not considered an option in the interest of turnaround time and sample usage. Data‐dependent acquisition (DDA) is a viable option for this type of analysis; however, it suffers from several drawbacks that make it less ideal for a compound‐specific quantitation method. Namely, it may tend to spend valuable instrument time performing MS2 scans on features that are not within one's prioritized compound set. However, a DDA method would allow for future investigation of other PFAS compounds outside of the analyte set of interest. The two more popular scan event choices for this type of method are parallel reaction monitoring (PRM) and the use of an inclusion list. PRM is essentially the equivalent of triple quadrupole MRM analysis, and for this reason it is an obvious and easy choice for this type of study. However, PRM analysis must be programmed by the user and does not use any type of data feedback. Thus, a PRM method has a tendency to use valuable analysis time searching for an analyte, even if it is not present. The user can program these PRM events into RT‐dependent windows such that they are only accessed for a particular molecule when it is expected to elute; however, under sub‐optimal chromatographic reproducibility (e.g., a new analytical column), this would lead to a molecule being incorrectly missed and that injection or multiple injections may need to be repeated. In this way a PRM method must be closely monitored (even after it is developed) to check that RTs are still accurate, and with the number of compounds that are typically being analyzed by these PFAS methods, this can be a very time‐consuming task.

**FIGURE 2:**
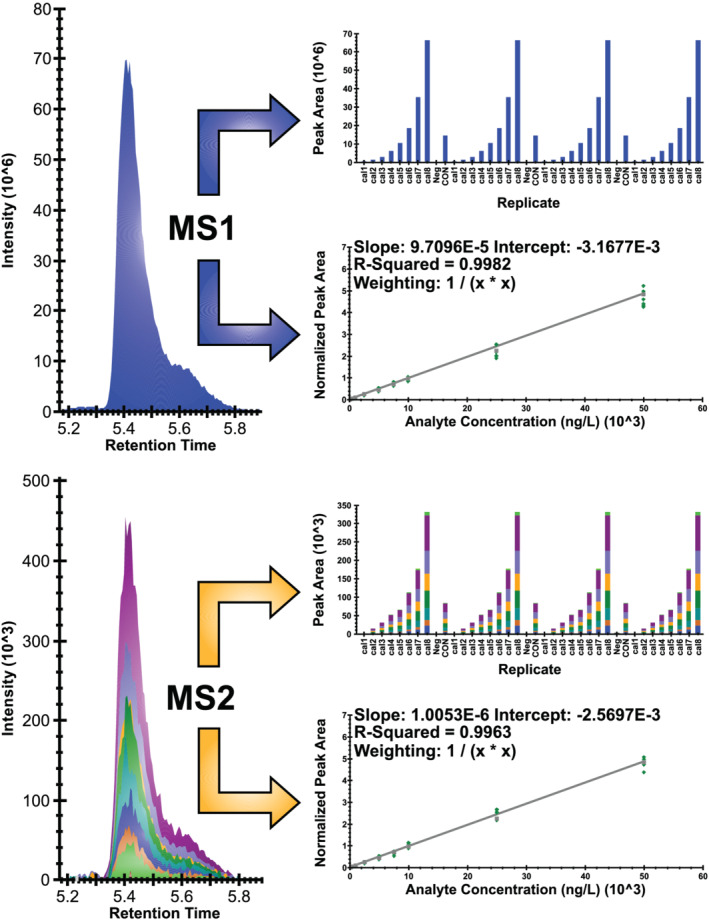
An example depiction of the data analysis workflow. MS1 and MS2 data (without dynamic exclusion) can be collected allowing for calibration in either the MS1 or MS2 dimension. Collection of MS2 data on high‐resolution equipment allows for dot product (dotp) qualitative verification [Color figure can be viewed at wileyonlinelibrary.com]

An inclusion list workflow operates from a user‐defined precursor mass list and triggers a MS2 scan when these precursor masses are detected in the MS1 space. This operates like a PRM where the MS1 data dictate the RT windows. This workflow can be used in conjunction with dynamic exclusion to control the number of times a detected MS1 mass is selected for subsequent fragmentation. Using a very strict dynamic exclusion (e.g., a dynamic exclusion window that is twice the width of a typical peak) will ensure a greater number of points across the MS1 peak and may provide more reproducible peak shapes for quantitation. A more relaxed dynamic exclusion (or no dynamic exclusion) can allow for peak area integration in the MS2 dimension, which can provide MS2 quantitation and/or better quality control metrics for verification of identification via MS2 fragmentation pattern matching performed via dot product (dotp) scores in Skyline.^12^ When an inclusion list scan event was used in this study in a strict manner and without dynamic exclusion, sufficient points across the MS1 peak were achieved in addition to chromatographic peaks in the MS2 dimension (Figure 2).

Most analytes in this method are detected in their de‐protonated form. The zwitterionic compounds (*N*‐AP‐FHxSA, *N*‐CMAmP‐6:2FOSA, and *N*‐TAmP‐FHxSA) are the only ones detected in their protonated, positive mode, forms. These compounds necessitate the use of a positive mode scan event to analyze for these compounds. Polarity switching like this is known to be a very slow process and can have a significant impact on the number of points across a peak. This can, in turn, affect the reproducibility of these signals and must be monitored carefully. HFPO‐DA presented a unique challenge as it predominates as its decarboxylated form as a result of in‐source fragmentation.^15^ For both the native and heavy isotope–labeled variants of this compound, the decarboxylated form was selected for MS1 peak integration.

A strength of this method is the inclusion of recently released standards from Fluoryx Labs (PEPA, PFO3OA, PFO4DA, and PFO5DoA), which were included as part of the validation. PEPA was observed in both the parent form and the decarboxylated form. The decarboxylated form, which produced a reproducible and more abundant signal, was chosen for quantitation. To the best of our knowledge, this is the first method to quantify any of these compounds using standards from an authentic supplier.

### 3.2 Data processing workflow development

Skyline software was used for data analysis.^14^ Skyline is a vendor‐neutral open‐source proteomics and metabolomics software for interrogating mass spectrometry data. Skyline recently incorporated tools to more easily allow for small molecule analysis.^16^ Quantitation can be performed on either MS1 or MS2 data, and in some cases the MS2 data may provide better sensitivity.^12^ However, for this workflow it was determined that there were no significant analyte sensitivity advantages when using MS2 data, and so quantitation was carried out using MS1 data for all analytes (data to be made publicly available via Panoramaweb once publication details are finalized).

**FIGURE 3:**
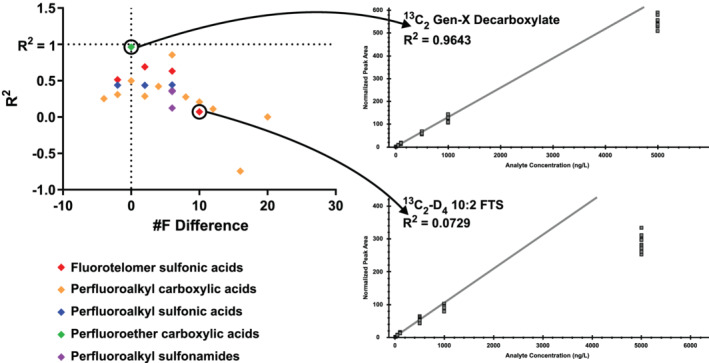
A scatterplot showing the effect of surrogate internal standard selection on the fit of the regression applied to the calibration curve [Color figure can be viewed at wileyonlinelibrary.com]

The choice of surrogate internal standard (when a matched standard is not available) is a very important one. Using a previously documented correlation between the number of fluorines and various solubility properties,^17^ surrogate internal standards were generally chosen based on the number of fluorine atoms compared to the unlabeled analyte. To show this relationship, PFO4DA was quantitated using 15 different internal standards. The *R* ^2^ value of the resulting calibration curve was plotted against the difference in the number of fluorines between the analyte and internal standard choice (Figure 3). This graph shows that selection of a surrogate internal standard is difficult to predict but that, in this case, the number of fluorines proved to be a good indicator. In some cases, the compound class ends up being a more accurate determinant of surrogacy potential.

### 3.3 Validation results

Ten replicate curves containing all analytes and internal standards were run. For each analyte, one curve was selected as the calibration curve and the rest were treated as quality control replicates to be tested against the curve. From these remaining nine replicates, at least seven had to be used to determine the appropriate validation metrics (Table 1 and supporting information). Using these criteria, MRLs and DLs were determined for all compounds. Limits were listed as both ng/L of the calibration stocks used (which were subsequently diluted slightly with internal standard) as well as picograms (pg) on column to avoid confusion. Oftentimes in literature, the limit values quoted are those of the 1 or 2 L solution that was pre‐concentrated down to 1 mL following extraction. Importantly, this method has no pre‐concentration or extraction steps.

**TABLE 1:** Table of minimum reporting limits (MRL), detection limit (DL), initial demonstration of precision (IDP), and initial demonstration of accuracy (IDA) for the compounds validated in this method

	Compound	MRL (ng/L)	MRL (pg on column)	DL (ng/L)	DL (pg on column)	IDP (%)^a^	IDA (%)^a^
1	4:2 FTS	10	0.9	2.44	0.220	1.44	111.43
2	6:2 FTS	10	0.9	2.76	0.249	7.66	112.22
3	8:2 FTS	10	0.9	3.26	0.294	8.61	102.66
4	10:2 FTS	5	0.45	1.58	0.142	2.84	112.47
5	NEtFOSAA	10	0.9	3.52	0.317	5.35	93.90
6	NMeFOSAA	10	0.9	3.25	0.292	7.71	91.61
7	FOSA	2	0.18	0.63	0.057	1.33	112.07
8	MeFOSA	2	0.18	0.70	0.063	2.50	102.00
9	FHxSA	2	0.18	0.81	0.073	5.85	110.70
10	FBSA	2	0.18	0.60	0.054	8.26	116.78
11	PFBS	5	0.45	1.61	0.145	10.93	106.92
12	PFPeS	2	0.18	0.32	0.029	3.41	110.28
13	PFHxS	10	0.9	2.93	0.264	7.86	122.19
14	PFHpS	2	0.18	0.70	0.063	12.74	117.85
15	PFOS	50	4.5	7.79	0.701	9.81	111.92
16	PFNS	2	0.18	0.48	0.043	2.65	104.26
17	PFDS	5	0.45	1.86	0.167	9.12	85.17
18	PFBA	50	4.5	19.08	1.717	1.08	113.30
19	PFPeA	10	0.9	3.91	0.352	7.09	104.00
20	PFHxA	5	0.45	1.74	0.157	8.34	117.01
21	PFHpA	5	0.45	1.56	0.140	6.80	111.06
22	PFOA	10	0.9	4.08	0.367	3.57	109.96
23	PFNA	5	0.45	1.60	0.144	1.45	111.32
24	PFDA	2	0.18	0.53	0.048	0.97	110.39
25	PFUdA	5	0.45	1.88	0.169	8.84	118.56
26	PFDoA	10	0.9	3.41	0.307	9.15	105.40
27	PFTrDA	10	0.9	2.80	0.252	8.59	99.14
28	PFTeDA	10	0.9	2.44	0.220	12.52	123.28
29	PFHxDA	100	9	32.37	2.913	10.99	96.89
30	PFODA	500	45	120.26	10.823	6.49	78.36
31	HFPO‐DA (Gen‐X) Decarboxylate	5	0.45	1.14	0.102	9.27	105.81
32	PFMOAA	500	45	138.66	12.480	6.02	87.60
33	NaDONA	2	0.18	0.80	0.072	3.75	105.52
34	PEPA	50	4.5	7.48	0.673	2.49	105.02
35	PFO3OA	50	4.5	12.46	1.121	2.05	106.30
36	PFO4DA	50	4.5	20.34	1.831	10.10	91.08
37	PFO5DoA	100	9	40.72	3.665	7.58	111.36
38	FHpPA	50	4.5	11.93	1.074	8.73	100.40
39	*N*‐AP‐FHxSA	10	0.9	2.08	0.187	16.84	115.62
40	*N*‐CMAmP‐6:2FOSA (6:2 FTAB)	10	0.9	4.26	0.383	12.49	95.69
41	*N*‐TAmP‐FHxSA	10	0.9	2.74	0.246	11.86	110.83
42	PS acid	5	0.45	0.70	0.063	2.65	114.58
43	Nafion by product 2	2	0.18	0.41	0.037	3.24	111.76
44	F53B Minor (11Cl‐PF3OUdS)	2	0.18	0.71	0.064	2.96	105.55
45	F53B Major (9Cl‐PF3ONS)	2	0.18	0.71	0.064	8.81	93.71

^a^ IDP and IDA measured at 500 ng/L.

Drinking water guidelines range from the low to mid‐parts per trillion (ng/L) range depending on the individual state.^8^ Most PFAS analysis workflows incorporate a significant pre‐concentration step using a weak anion exchange (WAX) SPE cartridge. This method is capable of achieving these required limits for drinking water analysis without the pre‐concentration step, thus saving time and money.

**FIGURE 4:**
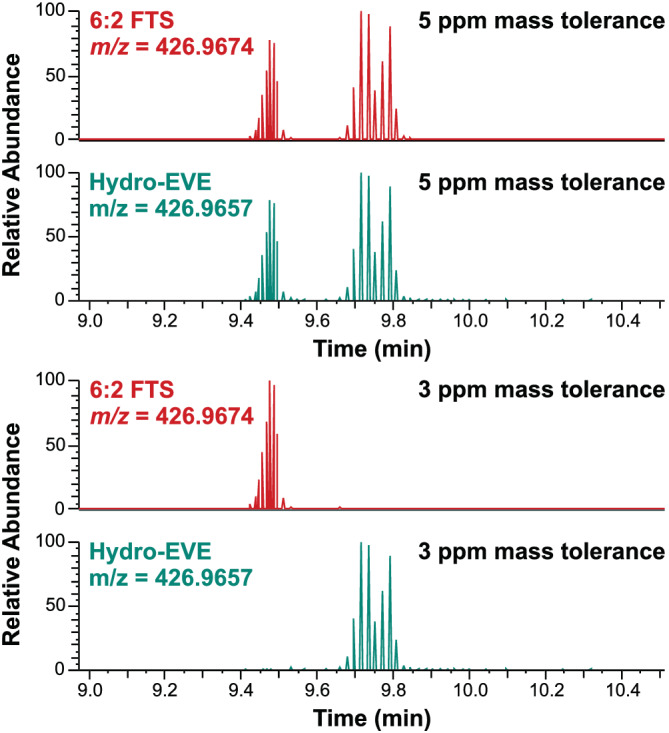
A plot showing the ability of this method to separate 6:2 FTS from Hydro‐EVE. Hydro‐EVE is not used in this method as there are no quality control standards available for this compound. The ability of this instrument to achieve sub‐3 ppm mass accuracy means these two compounds can be distinguished in the event that they are both present in an unknown sample [Color figure can be viewed at wileyonlinelibrary.com]

Although a triple quadrupole instrument could achieve lower limits in some cases, the numerous advantages of using a high resolving power instrument are evident. For example, the discrimination between 6:2 FTS (theoretical exact deprotonated mass = 426.9674) and Hydro‐EVE (theoretical exact deprotonated mass = 426.9657) has historically been very difficult (even on time‐of‐flight instrumentation) because of their similar precursor and fragment masses and a very similar retention profile on reverse phase LC column chemistries. Although no standard exists for Hydro‐EVE and it is therefore not in this method, using a high resolving power instrument affords the ability to separate these two species should they be present in an unknown sample. Although the Exploris 240 is specified to achieve sub 1 ppm mass accuracy using internal calibration, in practice it achieves less than 2‐3 ppm mass accuracy for these compounds. This value is still sufficient for discriminating this 3.98 ppm mass difference (Figure 4).

## 4 CONCLUSION

The method reported here was validated for 45 PFAS analytes and 23 PFAS internal standards according to certain metric such as MRL, DL, IDP, and IDA as they are described in EPA Method 537.1. Unlike most quantitative PFAS methods, this method is performed on a high resolving power Orbitrap Exploris system. In this way, labs that are interested in performing non‐targeted and targeted quantitative PFAS analysis can perform both on a single platform. In addition, we have described a workflow that incorporates a vendor‐neutral data analysis platform (Skyline) that allows researchers in this particularly collaborative field to more easily share results with other interested parties. This is also the first method to validate four newly available PFAS standards: PEPA, PFO3OA, PFO4DA, and PFO5DoA.

### PEER REVIEW

The peer review history for this article is available at https://publons.com/publon/10.1002/rcm.9295.

## Supporting information


**TABLE S1** Complete validation data for the high resolving power PFAS method presented in this manuscript. This method is validated across eight different chemical classes using recommended guidelines found in EPA Method 537.1 and Skyline data processing software

## Data Availability

The data that support the findings of this study are openly available on PanoramaWeb at https://panoramaweb.org/3NceL1.url.

## References

[1] Bartolome M , Gallego‐Pico A , Huetos O , Lucena MA , Castano A . A fast method for analysing six perfluoroalkyl substances in human serum by solid‐phase extraction on‐line coupled to liquid chromatography tandem mass spectrometry. Anal Bioanal Chem. 2016;408(8):2159‐2170. doi:10.1007/s00216-016-9319-0 26790871

[2] Ahrens L , Bundschuh M . Fate and effects of poly‐ and perfluoroalkyl substances in the aquatic environment: A review. Environ Toxicol Chem. 2014;33(9):1921‐1929. doi:10.1002/etc.2663 24924660

[3] EFSA Panel on Contaminants in the Food Chain (EFSA CONTAM Panel) , Schrenk D , Bignami M , et al. Risk to human health related to the presence of perfluoroalkyl substances in food. EFSA J. 2020;18(9):e06223. doi:10.2903/j.efsa.2020.6223 32994824 PMC7507523

[4] Loos R , Locoro G , Comero S , et al. Pan‐European survey on the occurrence of selected polar organic persistent pollutants in ground water. Water Res. 2010;44(14):4115‐4126. doi:10.1016/j.watres.2010.05.032 20554303

[5] Martin JW , Mabury SA , Solomon KR , Muir DC . Bioconcentration and tissue distribution of perfluorinated acids in rainbow trout (Oncorhynchus mykiss). Environ Toxicol Chem. 2003;22(1):196‐204. doi:10.1002/etc.5620220126 12503765

[6] Olsen GW , Mair DC , Lange CC , et al. Per‐ and polyfluoroalkyl substances (PFAS) in American red cross adult blood donors, 2000‐2015. Environ Res. 2017;157:87‐95. doi:10.1016/j.envres.2017.05.013 28528142

[7] Backe WJ , Day TC , Field JA . Zwitterionic, cationic, and anionic fluorinated chemicals in aqueous film forming foam formulations and groundwater from U.S. military bases by nonaqueous large‐volume injection HPLC–MS/MS. Environ Sci Technol. 2013;47(10):5226‐5234. doi:10.1021/es3034999 23590254

[8] Cordner A , De la Rosa VY , Schaider LA , Rudel RA , Richter L , Brown P . Guideline levels for PFOA and PFOS in drinking water: The role of scientific uncertainty, risk assessment decisions, and social factors. J Expo Anal Environ Epidemiol. 2019;29(2):157‐171. doi:10.1038/s41370-018-0099-9 PMC6455940 30622333

[9] Sun M , Arevalo E , Strynar M , et al. Legacy and emerging Perfluoroalkyl substances are important drinking water contaminants in the cape fear river watershed of North Carolina. Environ Sci Technol Lett. 2016;3(12):415‐419. doi:10.1021/acs.estlett.6b00398

[10] Washington JW , Rosal CG , McCord JP , et al. Nontargeted mass‐spectral detection of chloroperfluoropolyether carboxylates in New Jersey soils. Science. 2020;368(6495):1103‐1107. doi:10.1126/science.aba7127 32499438 PMC7814412

[11] McCord J , Strynar M . Identification of per‐ and Polyfluoroalkyl substances in the cape fear river by high resolution mass spectrometry and nontargeted screening. Environ Sci Technol. 2019;53(9):4717‐4727. doi:10.1021/acs.est.8b06017 30993978 PMC7478245

[12] Li ZC , Li YJ , Chen WJ , et al. Integrating MS1 and MS2 scans in high‐resolution parallel reaction monitoring assays for targeted metabolite quantification and dynamic C‐13‐labeling metabolism analysis. Anal Chem. 2017;89(1):877‐885. doi:10.1021/acs.analchem.6b03947 27966897

[13] Toprak UH , Gillet LC , Maiolica A , Navarro P , Leitner A , Aebersold R . Conserved peptide fragmentation as a benchmarking tool for mass spectrometers and a discriminating feature for targeted proteomics. Mol Cell Proteomics. 2014;13(8):2056‐2071. doi:10.1074/mcp.O113.036475 24623587 PMC4125737

[14] MacLean B , Tomazela DM , Shulman N , et al. Skyline: An open source document editor for creating and analyzing targeted proteomics experiments. Bioinformatics. 2010;26(7):966‐968. doi:10.1093/bioinformatics/btq054 20147306 PMC2844992

[15] Mullin L , Katz D , Riddell N , et al. Analysis of hexafluoropropylene oxide‐dimer acid (HFPO‐DA) by liquid chromatography‐mass spectrometry (LC‐MS): Review of current approaches and environmental levels. Trends Anal Chem. 2019;118:828‐839. doi:10.1016/j.trac.2019.05.015 PMC6733277 31501636

[16] Adams KJ , Pratt B , Bose N , et al. Skyline for small molecules: A unifying software package for quantitative metabolomics. J Proteome Res. 2020;19(4):1447‐1458. doi:10.1021/acs.jproteome.9b00640 31984744 PMC7127945

[17] Enders JR , O'Neill GM , Whitten JL , Muddiman DC . Understanding the electrospray ionization response factors of per‐ and poly‐fluoroalkyl substances (PFAS). Anal Bioanal Chem. 2022;414(3):1227‐1234. doi:10.1007/s00216-021-03545-8 34291300 PMC8727445

