# Supplementary material for: Development and validation of a high resolving power absolute quantitative per‐ and polyfluoroalkyl substances method incorporating Skyline data processing
Source: Rapid Commun Mass Spectrom. 2022 Mar 25;36(11):e9295. doi: 10.1002/rcm.9295 (PMC9287086; doi:10.1002/rcm.9295)
Supplement: Supplementary file 1 — TABLE S1 Complete validation data for the high resolving power PFAS method presented in this manuscript. This method is validated across eight different chemical classes using recommended guidelines found in EPA Method 537.1 and Skyline data processing software [file RCM-36-0-s001.pdf]

Development and Validation of a High-Resolving Power Absolute Quantitative PFAS method Incorporating Skyline Data Processing

Jeffrey R. Enders, Rebecca A. Weed, Emily Griffith, David C. Muddiman

Neat Validation 10/28/21

	Compound	Minimum Reporting Limit (MRL, ng/L)	Minimum Reporting Limit (MRL, pg on column)	Detection Limit (DL, ng/L)	Detection Limit (DL, pg on column)	Initial Demonstration of Precision (IDP)†	Initial Demonstration of Accuracy (IDA)†
1	**4:2 FTS**	10	0.9	2.44	0.220	1.44%	111.43%
2	**6:2 FTS**	10	0.9	2.76	0.249	7.66%	112.22%
3	**8:2 FTS**	10	0.9	3.26	0.294	8.61%	102.66%
4	**10:2 FTS**	5	0.45	1.58	0.142	2.84%	112.47%
5	**NEtFOSAA**	10	0.9	3.52	0.317	5.35%	93.90%
6	**NMeFOSAA**	10	0.9	3.25	0.292	7.71%	91.61%
7	**FOSA**	2	0.18	0.63	0.057	1.33%	112.07%
8	**MeFOSA**	2	0.18	0.70	0.063	2.50%	102.00%
9	**FHxSA**	2	0.18	0.81	0.073	5.85%	110.70%
10	**FBSA**	2	0.18	0.60	0.054	8.26%	116.78%
11	**PFBS**	5	0.45	1.61	0.145	10.93%	106.92%
12	**PFPeS**	2	0.18	0.32	0.029	3.41%	110.28%
13	**PFHxS**	10	0.9	2.93	0.264	7.86%	122.19%
14	**PFHpS**	2	0.18	0.70	0.063	12.74%	117.85%
15	**PFOS**	50	4.5	7.79	0.701	9.81%	111.92%
16	**PFNS**	2	0.18	0.48	0.043	2.65%	104.26%
17	**PFDS**	5	0.45	1.86	0.167	9.12%	85.17%
18	**PFBA**	50	4.5	19.08	1.717	1.08%	113.30%
19	**PFPeA**	10	0.9	3.91	0.352	7.09%	104.00%
20	**PFHxA**	5	0.45	1.74	0.157	8.34%	117.01%
21	**PFHpA**	5	0.45	1.56	0.140	6.80%	111.06%
22	**PFOA**	10	0.9	4.08	0.367	3.57%	109.96%
23	**PFNA**	5	0.45	1.60	0.144	1.45%	111.32%
24	**PFDA**	2	0.18	0.53	0.048	0.97%	110.39%
25	**PFUdA**	5	0.45	1.88	0.169	8.84%	118.56%
26	**PFDoA**	10	0.9	3.41	0.307	9.15%	105.40%
27	**PFTrDA**	10	0.9	2.80	0.252	8.59%	99.14%
28	**PFTeDA**	10	0.9	2.44	0.220	12.52%	123.28%
29	**PFHxDA**	100	9	32.37	2.913	10.99%	96.89%
30	**PFODA**	500	45	120.26	10.823	6.49%	78.36%
31	**HFPO-DA (Gen-X) Decarboxylate**	5	0.45	1.14	0.102	9.27%	105.81%
32	**PFMOAA**	500	45	138.66	12.480	6.02%	87.60%
33	**NaDONA**	2	0.18	0.80	0.072	3.75%	105.52%
34	**PEPA**	50	4.5	7.48	0.673	2.49%	105.02%
35	**PFO3OA**	50	4.5	12.46	1.121	2.05%	106.30%
36	**PFO4DA**	50	4.5	20.34	1.831	10.10%	91.08%
37	**PFO5DoA**	100	9	40.72	3.665	7.58%	111.36%
38	**FHpPA**	50	4.5	11.93	1.074	8.73%	100.40%
39	**N-AP-FHxSA**	10	0.9	2.08	0.187	16.84%	115.62%
40	**N-CMAmP-6:2FOSA (6:2 FTAB)**	10	0.9	4.26	0.383	12.49%	95.69%
41	**N-TAmP-FHxSA**	10	0.9	2.74	0.246	11.86%	110.83%
42	**PS Acid**	5	0.45	0.70	0.063	2.65%	114.58%
43	**Nafion byproduct 2**	2	0.18	0.41	0.037	3.24%	111.76%
44	**F53B Minor (11Cl-PF3OUdS)**	2	0.18	0.71	0.064	2.96%	105.55%
45	**F53B Major (9Cl-PF3ONS)**	2	0.18	0.71	0.064	8.81%	93.71%

† IDP and IDA measured at 500 ng/L

Assay Name:	SRP PFAS	New Method Validation			
Instrument:	Exploris 240	Regression:	Linear	Weighting:	$1/x^2$
Analyte:	4:2 FTS				

DAY 1

Calibrators (ng/L)		~~1~~	~~2~~	5	10	50	100	500	1,000	5,000	10,000		
Date	10/27/2021												
R^2	0.9925	1											

MRL =	10	ng/L	IDP =	1.44%	@ 500 ng/L
DL =	2.44	ng/L	IDA =	111.43%	@ 500 ng/L

Rep		1	2	5	10	50	100	500	1,000	Neg	5,000	Neg	10,000	Neg	CON 800
1					9.68	51.04	94.08	558.74	1,054.66	0.40	5,022.07	0.29	9,247.54	1.24	796.69
2					9.73	50.21	97.75	558.81	1,050.08	0.46	4,966.68	0.91	9,245.84	0.15	796.13
3					10.97	55.81	95.39	567.19	1,074.27	0.69	4,927.23	1.09	9,408.45	(0.05)	812.22
4					11.86	49.86	95.73	562.75	1,052.07	0.75	5,003.34	0.76	9,334.81	1.14	816.22
5					10.15	49.15	84.50	564.83	1,017.26	0.74	4,991.03	1.16	9,383.75	1.48	801.07
6					9.90	51.68	94.31	553.71	1,060.64	4.87	4,988.58	0.72	9,288.41	0.67	795.36
7					10.28	51.71	76.80	543.19	1,038.39	2.39	5,253.47	1.41	9,940.67	2.59	823.10
8					10.44	49.95	77.93	549.51	1,057.90	0.81	4,947.56	0.48	9,256.21	1.19	800.91
9					9.30	51.30	81.28	555.53	1,047.04	0.52	4,964.81	2.07	8,732.96	0.80	812.88
	N=				9	9	9	9	9	9	9	9	9	9	9
	t value				3.355	3.355	3.355	3.355	3.355	3.355	3.355	3.355	3.355	3.355	3.355
**Metric**	**Criteria**														
Mean					10.26	51.19	88.64	557.14	1050.26	1.29	5007.20	0.99	9315.40	1.02	806.06
Std Dev					0.73	1.84	7.94	7.18	14.93	1.39	91.22	0.50	290.13	0.73	9.61
RSD	< 20%				7.29%			1.44%							
Accuracy	± 30%				102.57%			111.43%							
HR_{PIR}					2.72										
Upper PIR	≤ 150%				130%										
Lower PIR	≥ 50%				75%										

Assay Name:	SRP PFAS	New Method Validation			
Instrument:	Exploris 240	Regression:	Linear	Weighting:	$1/x^2$
Analyte:	6:2 FTS				

DAY 1

Calibrators (ng/L)	~~1~~	~~2~~	~~5~~	10	50	100	500	1,000	5,000	~~10000~~		

Date 10/27/2021

R^2 0.997 1

MRL =	10	ng/L	IDP =	7.66%	@ 500 ng/L
DL =	2.76	ng/L	IDA =	112.22%	@ 500 ng/L

Rep		1	2	5	10	50	100	500	1,000	Neg	5,000	Neg	10,000	Neg	CON 800
1					10.24	48.21	96.58	528.63	1,263.97	1.54	6,943.30	4.39	13,024.60	1.72	760.01
2					10.70	60.12	81.83	510.28	953.78	0.93	4,852.90	0.93	10,747.48	2.61	748.12
3					11.79	54.79	82.43	530.52	1,276.18	2.75	6,282.66	1.77	10,351.19	0.93	895.00
4					12.45	62.04	117.37	571.91	976.37	9.14	5,769.21	2.03	11,500.38	2.09	890.26
5					11.82	58.47	81.02	613.66	941.50	0.93	4,924.17	1.73	13,715.00	1.79	760.66
6					12.79	49.69	119.88	548.65	963.09	0.93	4,702.97	0.93	8,705.88	2.16	977.92
7					11.67	55.91	107.88	*717.4	1,244.69	1.20	4,689.41	0.93	8,848.40	1.35	801.91
8					11.28	50.83	93.84	559.60	956.43	1.75	4,893.61	2.63	10,673.36	1.91	924.32
9					*8.99	46.36	109.64	625.42	965.32	0.93	6,208.28	0.93	11,395.83	1.98	838.39
	N=				8	9	9	8	9	9	9	9	9	9	9
	t value				3.499	3.355	3.355	3.499	3.355	3.355	3.355	3.355	3.355	3.355	3.355
**Metric**	**Criteria**														
Mean					11.59	54.05	98.94	561.08	1060.15	2.23	5474.06	1.81	10995.79	1.84	844.07
Std Dev					0.79	5.24	14.49	38.32	142.93	2.51	794.01	1.08	1576.24	0.46	77.55
RSD	< 20%				7.90%			7.66%							
Accuracy	± 30%				115.93%			112.22%							
HR_{PIR}					3.11										
Upper PIR	≤ 150%				147%										
Lower PIR	≥ 50%				85%										

Assay Name:	SRP PFAS	New Method Validation			
Instrument:	Exploris 240	Regression:	Linear	Weighting:	$1/x^2$
Analyte:	8:2 FTS				

DAY 1

Calibrators (ng/L)	~~1~~	~~2~~	~~5~~	10	50	100	500	1,000	5,000	10,000		

Date 10/27/2021

R^2 0.9948 9

MRL =	10	ng/L	IDP =	8.61%	@ 500 ng/L
DL =	3.26	ng/L	IDA =	102.66%	@ 500 ng/L

Rep		1	2	5	10	50	100	500	1,000	Neg	5,000	Neg	10,000	Neg	CON 800
1					*14.23	50.24	101.00	606.68	1,229.47	1.29	5,575.13	0.67	10,477.90	1.46	960.44
2					10.80	54.12	89.29	*647.22	1,157.62	0.57	5,395.75	9.29	10,226.88	2.22	886.42
3					9.58	58.20	94.25	*674.31	1,128.95	0.57	5,855.62	1.41	10,315.92	2.76	933.72
4					*12.21	51.24	84.69	492.51	919.16	3.61	4,209.29	0.57	7,731.34	1.68	689.44
5					8.81	44.63	77.54	515.03	1,013.01	13.22	5,101.04	1.66	9,823.79	2.28	704.60
6					*12.05	54.25	100.78	480.69	973.76	1.46	5,767.33	2.39	11,011.75	2.06	942.59
7					*7.1	56.84	80.35	496.70	1,138.67	0.57	5,369.03	0.57	10,148.66	2.04	704.43
8					9.10	46.71	75.44	488.23	987.49	0.57	5,477.12	2.37	8,083.79	1.27	724.90
9					9.07	59.52	112.03	*636.48	1,165.44	3.03	5,882.92	1.50	9,931.49	4.26	902.85
	N=				5	9	9	6	9	9	9	9	9	9	9
	t value				4.604	3.355	3.355	4.032	3.355	3.355	3.355	3.355	3.355	3.355	3.355
**Metric**	**Criteria**														
Mean					9.47	52.86	90.60	513.31	1079.29	2.77	5403.69	2.27	9750.17	2.23	827.71
Std Dev					0.71	4.80	11.67	43.06	100.94	3.85	485.38	2.57	1039.13	0.84	111.15
RSD	< 20%				7.09%			8.61%							
Accuracy	± 30%				94.72%			102.66%							
HR_{PIR}					3.92										
Upper PIR	≤ 150%				134%										
Lower PIR	≥ 50%				56%										

**Assay Name:**	**SRP PFAS**	**New Method Validation**	
**Instrument:**	**Exploris 240**	**Regression:** Linear	**Weighting:** $1/x^2$
**Analyte:**	**10:2 FTS**		

DAY 1

**Calibrators (ng/L)**	**~~1~~**	**~~2~~**	**5**	**10**	**50**	**100**	**500**	**1,000**	**5,000**	**10,000**		

Date 10/27/2021

R^2 0.9995 4

**MRL =**	**5**	**ng/L**	**IDP =**	**2.84%**	**@ 500 ng/L**
**DL =**	**1.58**	**ng/L**	**IDA =**	**112.47%**	**@ 500 ng/L**

Rep		**1**	**2**	**5**	**10**	**50**	**100**	**500**	**1,000**	**Neg**	**5,000**	**Neg**	**10,000**	**Neg**	**CON 800**
1				3.86	8.89	44.41	87.39	550.19	1,038.37	3.10	5,264.08	0.83	9,499.48	1.47	840.49
2				4.00	9.32	46.37	77.60	552.55	1,028.92	1.04	5,243.32	7.10	9,318.54	2.26	869.81
3				4.78	12.25	55.68	71.32	547.44	1,046.37	0.55	5,131.28	1.47	9,680.97	2.45	860.88
4				4.72	10.63	49.39	90.66	549.91	1,047.89	4.52	5,211.03	0.41	10,105.12	2.57	809.45
5				4.82	9.93	49.34	86.30	565.43	952.56	(0.58)	5,167.73	1.79	9,889.12	4.02	841.25
6				4.04	7.35	55.71	101.26	573.36	1,037.22	(0.72)	5,145.51	1.58	10,090.80	2.60	848.86
7				4.06	9.06	57.44	93.44	593.13	1,030.56	(0.60)	5,221.59	3.37	10,177.63	0.85	840.54
8				*6.03	7.84	52.67	94.22	571.83	1,035.33	3.36	5,279.95	1.98	10,456.92	3.64	840.61
9				5.12	10.51	52.33	96.72	557.44	1,023.65	(0.06)	5,432.49	0.90	10,111.51	4.28	833.68
	N=			8	9	9	9	9	9	9	9	9	9	9	9
	t value			3.499	3.355	3.355	3.355	3.355	3.355	3.355	3.355	3.355	3.355	3.355	3.355
**Metric**	**Criteria**														
Mean				4.43	9.53	51.48	88.77	562.36	1026.76	1.18	5233.00	2.16	9925.57	2.68	842.84
Std Dev				0.45	1.41	4.19	8.89	14.19	27.25	1.87	85.60	1.92	341.76	1.07	16.01
RSD	< 20%			9.04%				2.84%							
Accuracy	± 30%			88.50%				112.47%							
HR_{PIR}				1.78											
Upper PIR	≤ 150%			124%											
Lower PIR	≥ 50%			53%											

**Assay Name:**	**SRP PFAS**	**New Method Validation**	
**Instrument:**	**Exploris 240**	**Regression:** **Linear**	**Weighting:** **$1/x^2$**
**Analyte:**	**NEtFOSAA**		

DAY 1

**Calibrators (ng/L)**	~~**1**~~	~~**2**~~	~~**5**~~	**10**	**50**	**100**	**500**	**1,000**	**5,000**	**10,000**		

Date **10/27/2021**

R^2 **0.9991** **10**

**MRL =**	**10**	**ng/L**	**IDP =**	**5.35%**	**@ 500 ng/L**
**DL =**	**3.52**	**ng/L**	**IDA =**	**93.90%**	**@ 500 ng/L**

Rep		**1**	**2**	**5**	**10**	**50**	**100**	**500**	**1,000**	**Neg**	**5,000**	**Neg**	**10,000**	**Neg**	**CON 800**
1					10.42	43.71	79.69	430.13	842.13	3.69	4,619.90	10.34	9,029.76	10.86	737.20
2					*12.04	45.71	79.51	506.85	888.38	3.62	4,833.08	9.02	8,558.18	13.54	761.00
3					10.5	51.91	83.62	454.51	931.96	4.96	4,712.66	6.62	9,302.95	10.28	753.96
4					*11.88	48.71	71.12	431.33	763.89	3.34	3,842.19	6.19	7,671.10	9.60	673.43
5					*7.54	39.44	65.01	463.72	812.89	5.18	4,764.25	7.47	11,666.42	11.59	646.69
6					8.8	44.73	75.75	474.95	759.06	4.40	4,759.01	8.37	9,992.96	11.31	729.71
7					9.5	49.55	76.51	498.12	930.50	3.85	4,370.89	6.47	8,937.06	9.43	629.04
8					*7.31	37.42	67.16	466.15	767.71	4.04	4,341.13	6.60	8,747.83	9.75	596.84
9					8.71	45.11	91.13	499.70	852.40	4.41	4,411.39	7.71	9,890.27	12.63	769.83
		N=			5	9	9	9	9	9	9	9	9	9	9
	t value				4.604	3.355	3.355	3.355	3.355	3.355	3.355	3.355	3.355	3.355	3.355
**Metric**	**Criteria**														
Mean					9.59	45.14	76.61	469.50	838.77	4.17	4517.17	7.64	9310.73	11.00	699.74
Std Dev					0.76	4.38	7.69	26.73	64.50	0.59	295.76	1.31	1061.49	1.34	60.55
RSD	< 20%				7.65%			5.35%							
Accuracy	± 30%				95.86%			93.90%							
HR_{PIR}					4.22										
Upper PIR	≤ 150%				138%										
Lower PIR	≥ 50%				54%										

**Assay Name:**	**SRP PFAS**	**New Method Validation**					
**Instrument:**	**Exploris 240**	**Regression:**	**Linear**	**Weighting:**	**$1/x^2$**		
**Analyte:**	**NMeFOSAA**						

DAY 1

**Calibrators (ng/L)**	~~**1**~~	~~**2**~~	~~**5**~~	**10**	**50**	**100**	**500**	**1,000**	**5,000**	**10,000**		
**Date**	**10/27/2021**											
**R^2**	**0.9993**	**10**										

**MRL =**	**10**	**ng/L**	**IDP =**	**7.71%**	**@ 500 ng/L**
**DL =**	**3.25**	**ng/L**	**IDA =**	**91.61%**	**@ 500 ng/L**

Rep		**1**	**2**	**5**	**10**	**50**	**100**	**500**	**1,000**	**Neg**	**5,000**	**Neg**	**10,000**	**Neg**	**CON 800**
1					10.59	51.12	75.02	458.78	875.15	4.69	5,115.29	7.84	9,659.89	13.08	738.18
2					10.94	48.64	78.38	541.52	911.26	4.24	4,974.32	10.99	8,183.90	11.74	824.26
3					*13.86	51.37	83.83	452.47	871.28	4.71	5,097.00	9.16	9,868.17	12.72	763.81
4					11.22	47.04	71.67	425.32	751.06	4.84	3,278.16	6.43	8,198.02	9.47	569.29
5					9.34	40.48	63.98	423.14	800.99	5.94	4,479.45	8.63	9,403.14	12.31	642.60
6					12.01	47.49	82.17	456.00	756.83	3.17	5,264.43	10.23	8,431.06	12.06	779.30
7					10.65	38.82	78.43	450.39	948.56	4.76	4,282.61	8.30	8,578.95	11.42	638.84
8					*8.45	36.87	64.80	412.42	768.01	4.68	4,632.24	6.78	8,167.63	10.15	510.49
9					12.12	46.56	96.19	502.26	837.10	3.17	4,466.26	8.89	10,559.14	14.39	841.13
	N=				7	9	9	9	9	9	9	9	9	9	9
	t value				3.707	3.355	3.355	3.355	3.355	3.355	3.355	3.355	3.355	3.355	3.355
**Metric**	**Criteria**														
Mean					10.98	45.38	77.16	458.03	835.58	4.47	4621.08	8.58	9005.54	11.93	700.88
Std Dev					0.88	5.03	9.40	38.57	67.03	0.81	574.61	1.39	835.69	1.40	109.14
RSD	< 20%				8.76%			7.71%							
Accuracy	± 30%				109.81%			91.61%							
HR_{PIR}					3.71										
Upper PIR	≤ 150%				147%										
Lower PIR	≥ 50%				73%										

**Assay Name:**	**SRP PFAS**	**New Method Validation**	
**Instrument:**	**Exploris 240**	**Regression:** **Linear**	**Weighting:** $1/x^2$
**Analyte:**	**FOSA**		

DAY 1

**Calibrators (ng/L)**		~~1~~	**2**	**5**	**10**	**50**	**100**	**500**	**1,000**	**5,000**	**10,000**		
**Date**	**10/27/2021**												
**R^2**	**0.9982**	**1**											

**MRL =**	**2**	**ng/L**	**IDP =**	**1.33%**	**@ 500 ng/L**
**DL =**	**0.63**	**ng/L**	**IDA =**	**112.07%**	**@ 500 ng/L**

Rep		**1**	**2**	**5**	**10**	**50**	**100**	**500**	**1,000**	**Neg**	**5,000**	**Neg**	**10,000**	**Neg**	**CON 800**
1			*1.6	4.94	9.72	49.27	80.17	554.20	995.56	0.39	5,012.63	1.88	9,150.50	0.58	846.08
2			2.21	5.03	9.36	51.09	77.18	562.36	1,039.32	0.39	4,993.28	0.39	9,542.11	0.76	843.31
3			2.33	5.18	10.4	56.24	81.44	552.55	1,053.93	1.18	5,485.17	0.39	9,909.70	0.58	863.35
4			1.81	4.89	10.96	52.66	91.04	557.30	1,045.32	6.15	5,147.23	0.52	9,593.18	0.79	830.51
5			2.01	5.29	9.76	49.81	86.18	569.40	975.86	0.52	5,120.35	0.66	9,520.82	1.37	826.86
6			2	5	10.92	53.87	99.26	572.30	1,045.39	0.39	5,066.15	0.78	9,844.29	1.20	840.98
7			*2.64	5.44	10.38	54.24	92.56	561.87	1,018.04	0.60	5,008.98	0.71	9,753.16	0.39	847.50
8			1.88	4.74	10.17	51.38	102.06	560.22	1,026.07	0.39	5,089.47	0.62	10,096.42	1.47	829.92
9			1.95	5.07	8.74	50.04	97.99	552.74	1,006.38	0.39	5,248.09	0.56	10,048.71	0.78	827.36
	N=		7	9	9	9	9	9	9	9	9	9	9	9	9
	t value		3.707	3.355	3.355	3.355	3.355	3.355	3.355	3.355	3.355	3.355	3.355	3.355	3.355
**Metric**	**Criteria**														
Mean			2.03	5.06	10.05	52.07	89.76	560.33	1022.87	1.16	5130.15	0.72	9717.65	0.88	839.54
Std Dev			0.17	0.20	0.68	2.22	8.50	6.64	24.74	1.78	146.56	0.43	281.65	0.36	11.41
RSD	< 20%		8.47%					1.33%							
Accuracy	± 30%		101.36%					112.07%							
HR_{PIR}			0.72												
Upper PIR	≤ 150%		137%												
Lower PIR	≥ 50%		65%												

**Assay Name:**	**SRP PFAS**	**New Method Validation**				
**Instrument:**	**Exploris 240**	**Regression:**	**Linear**	**Weighting:**	**$1/x^2$**	
**Analyte:**	**MeFOSA**					

DAY 1

**Calibrators (ng/L)**	**~~1~~**	**2**	**5**	**10**	**50**	**100**	**500**	**1,000**	**5,000**	**10,000**
**Date**	**10/27/2021**									
**R^2**	**0.9975**	**1**								

**MRL =**	**2**	**ng/L**	**IDP =**	**2.50%**	**@ 500 ng/L**
**DL =**	**0.70**	**ng/L**	**IDA =**	**102.00%**	**@ 500 ng/L**

Rep		**1**	**2**	**5**	**10**	**50**	**100**	**500**	**1,000**	**Neg**	**5,000**	**Neg**	**10,000**	**Neg**	**CON 800**
1			2.25	5.39	10.42	49.69	96.36	519.69	1,016.96	0.56	5,837.31	2.95	10,434.97	1.05	832.65
2			2.09	6.11	14.37	65.98	92.50	512.29	938.26	0.47	5,455.40	0.43	10,925.97	0.78	771.43
3			1.75	7.00	11.57	58.82	77.44	494.26	937.47	1.26	4,574.18	0.31	9,336.77	0.88	776.59
4			2.09	5.22	10.01	51.60	86.50	491.79	924.16	2.29	5,273.40	0.42	11,958.80	0.87	741.15
5			*2.61	5.43	10.70	49.23	84.49	503.44	848.59	0.85	5,852.90	0.80	11,854.07	1.13	752.70
6			*2.59	4.08	11.01	51.50	96.68	515.94	929.99	0.95	4,573.46	1.07	9,395.91	0.75	743.32
7			*2.47	4.55	10.24	51.03	86.77	518.98	906.79	0.24	4,488.25	0.59	9,097.81	0.67	738.80
8			1.81	5.59	10.51	51.36	95.14	501.35	895.91	1.26	4,634.83	1.36	11,029.17	2.10	774.99
9			1.96	5.29	10.59	48.53	100.27	532.16	1,063.46	0.95	5,336.34	0.72	12,639.73	0.76	921.73
		N=	6	9	9	9	9	9	9	9	9	9	9	9	9
	t value		4.032	3.355	3.355	3.355	3.355	3.355	3.355	3.355	3.355	3.355	3.355	3.355	3.355
**Metric**	**Criteria**														
Mean			1.99	5.41	11.05	53.08	90.68	509.99	940.18	0.98	5114.01	0.96	10741.47	1.00	783.71
Std Dev			0.17	0.79	1.25	5.36	6.91	12.49	60.49	0.57	523.38	0.77	1202.74	0.41	55.92
RSD	< 20%		8.62%					2.50%							
Accuracy	± 30%		99.58%					102.00%							
HR_{PIR}			0.81												
Upper PIR	≤ 150%		140%												
Lower PIR	≥ 50%		59%												

**Assay Name:**	**SRP PFAS**	**New Method Validation**			
**Instrument:**	**Exploris 240**	**Regression:**	**Linear**	**Weighting:**	**$1/x^2$**
**Analyte:**	**FHxSA**				
**Internal Standard**	**PFBS**				

DAY 1

**Calibrators (ng/L)**	~~**1**~~	**2**	**5**	**10**	**50**	**100**	**500**	**1,000**	**5,000**	**10,000**		

Date 10/27/2021

R^2 0.997 1

**MRL =**	**2**	**ng/L**	**IDP =**	**5.85%**	**@ 500 ng/L**
**DL =**	**0.81**	**ng/L**	**IDA =**	**110.70%**	**@ 500 ng/L**

Rep		**1**	**2**	**5**	**10**	**50**	**100**	**500**	**1,000**	**Neg**	**5,000**	**Neg**	**10,000**	**Neg**	**CON 800**
1			*1.55	4.6	10.10	46.48	73.13	559.84	971.11	0.31	5,283.97	0.51	11,676.07	0.31	851.52
2			1.8	4.4	9.26	51.10	71.90	549.01	1,081.76	0.31	6,048.35	0.31	9,904.90	0.64	832.79
3			2.11	4.39	8.81	53.63	82.47	503.25	1,123.41	0.31	5,495.82	0.31	12,370.66	0.31	868.60
4			2.24	4.44	10.45	47.81	94.12	571.66	1,133.46	1.51	6,281.83	0.31	10,497.12	0.38	885.92
5			*1.55	4.18	8.45	47.98	93.22	520.47	1,100.72	0.31	6,453.51	0.31	12,572.32	0.31	885.53
6			1.63	4.64	10.14	52.70	103.87	587.73	1,003.13	0.31	6,259.24	0.31	10,957.09	0.49	789.38
7			*2.5	5.01	8.39	53.11	101.70	524.99	1,130.59	0.31	6,366.91	0.31	10,796.31	0.31	885.55
8			2.01	4.5	8.98	54.85	91.02	587.68	1,119.40	0.31	6,256.00	0.60	10,773.09	0.31	865.35
9			2.01	4.55	9.99	47.23	101.86	576.93	1,126.05	0.31	6,434.10	0.31	12,184.93	0.31	879.13
		N=	6	9	9	9	9	9	9	9	9	9	9	9	9
	t value		4.032	3.355	3.355	3.355	3.355	3.355	3.355	3.355	3.355	3.355	3.355	3.355	3.355
**Metric**	**Criteria**														
Mean			1.97	4.52	9.40	50.54	90.37	553.51	1087.74	0.44	6097.75	0.36	11303.61	0.37	860.42
Std Dev			0.20	0.21	0.74	3.00	11.38	29.26	56.41	0.38	397.79	0.10	877.46	0.11	30.26
RSD	< 20%		10.00%					5.85%							
Accuracy	± 30%		98.33%					110.70%							
HR_{PIR}			0.94												
Upper PIR	≤ 150%		145%												
Lower PIR	≥ 50%		51%												

**Assay Name:**	**SRP PFAS**	**New Method Validation**			
**Instrument:**	**Exploris 240**	**Regression:**	**Linear**	**Weighting:**	**$1/x^2$**
**Analyte:**	**FBSA**				
**Internal Standard**	**PFBS**				

DAY 1

**Calibrators (ng/L)**	~~**1**~~	**2**	**5**	**10**	**50**	**100**	**500**	**1,000**	**5,000**	**10,000**		

Date **10/27/2021**

R^2 **0.99** 1

**MRL =**	**2**	**ng/L**	**IDP =**	**8.26%**	**@ 500 ng/L**
**DL =**	**0.60**	**ng/L**	**IDA =**	**116.78%**	**@ 500 ng/L**

Rep		**1**	**2**	**5**	**10**	**50**	**100**	**500**	**1,000**	**Neg**	**5,000**	**Neg**	**10,000**	**Neg**	**CON 800**
1			1.86	4.81	9.82	44.57	78.56	604.04	1,032.32	0.73	6,262.78	0.73	12,453.46	0.89	768.49
2			2.21	6.74	9.81	58.19	70.84	537.35	971.50	0.73	5,660.05	0.73	10,522.95	0.73	748.61
3			2.16	4.31	10.32	48.36	83.03	594.12	1,343.76	0.73	6,401.12	0.73	12,024.19	0.98	1,027.07
4			1.97	5.38	12.01	61.69	115.83	*697.95	1,192.76	1.30	5,338.27	0.73	11,051.23	0.73	778.00
5			2.07	4.27	10.3	44.44	97.64	549.61	1,226.88	0.73	6,678.02	0.73	13,206.86	0.73	782.12
6			*3.13	6.49	11.86	51.67	111.89	531.22	944.90	0.73	5,535.82	0.73	10,561.45	0.73	867.34
7			2.09	6.53	12.75	64.01	117.12	*658.57	1,337.34	0.73	5,718.71	0.73	9,841.56	0.73	1,063.69
8			2.44	5.92	11.56	60.45	85.97	637.13	1,072.49	0.73	6,381.92	0.91	9,472.76	0.73	778.67
9			1.93	6.7	11.47	52.63	100.21	633.66	1,127.44	0.73	6,242.72	0.73	12,495.63	0.73	920.03
	N=		8	9	9	9	9	7	9	9	9	9	9	9	9
	t value		3.499	3.355	3.355	3.355	3.355	3.707	3.355	3.355	3.355	3.355	3.355	3.355	3.355
**Metric**	**Criteria**														
Mean			2.09	5.68	11.10	54.00	95.68	583.88	1138.82	0.79	6024.38	0.75	11292.23	0.78	859.34
Std Dev			0.17	0.96	1.00	6.98	16.05	41.30	138.86	0.18	439.33	0.06	1229.14	0.09	112.17
RSD	< 20%		8.57%					8.26%							
Accuracy	± 30%		104.56%					116.78%							
HR_{PIR}			0.67												
Upper PIR	≤ 150%		138%												
Lower PIR	≥ 50%		71%												

**Assay Name:**	**SRP PFAS**		**New Method Validation**
**Instrument:**	**Exploris 240**	**Regression:** Linear	**Weighting:** $1/x^2$
**Analyte:**	**PFBS**		

DAY 1

**Calibrators (ng/L)**	~~1~~	~~2~~	5	10	50	100	500	1,000	5,000	10,000		

Date 10/27/2021

R^2 0.9983 1

**MRL =**	**5**	**ng/L**	**IDP =**	**10.93%**	**@ 500 ng/L**
**DL =**	**1.61**	**ng/L**	**IDA =**	**106.92%**	**@ 500 ng/L**

Rep		1	2	5	10	50	100	500	1,000	Neg	5,000	Neg	10,000	Neg	CON 800
1				4.82	9.65	51.93	86.45	616.37	1,147.22	(0.40)	5,553.75	2.09	9,749.46	0.10	864.85
2				4.96	10.05	54.25	79.21	601.11	1,076.18	(0.68)	5,117.56	(0.26)	10,078.64	(0.06)	881.97
3				4.80	9.69	50.73	68.41	493.81	882.33	0.18	5,174.33	(0.46)	8,742.30	(0.59)	755.38
4				4.06	8.23	42.53	82.32	478.41	992.13	3.17	5,275.85	(0.52)	10,245.60	(0.17)	686.45
5				5.11	10.39	52.36	85.22	536.98	887.11	(0.73)	5,432.95	(0.31)	10,317.57	(0.19)	882.13
6				4.19	8.35	42.52	79.88	468.10	939.00	(0.84)	4,767.08	(0.61)	9,043.78	(0.51)	747.87
7				4.09	8.11	42.49	86.68	491.97	858.70	(0.61)	4,730.09	(0.93)	8,588.58	(0.69)	816.44
8				3.58	8.39	44.83	90.44	522.54	980.61	(0.22)	4,295.16	(0.38)	9,737.87	(0.10)	878.85
9				4.35	8.95	46.12	95.71	602.15	1,150.54	(0.45)	5,546.22	(0.74)	9,516.42	(0.21)	875.23
	N=			9	9	9	9	9	9	9	9	9	9	9	9
	t value			3.355	3.355	3.355	3.355	3.355	3.355	3.355	3.355	3.355	3.355	3.355	3.355
**Metric**	**Criteria**														
Mean				4.44	9.09	47.53	83.81	534.60	990.42	-0.06	5099.22	-0.24	9557.80	-0.27	821.02
Std Dev				0.48	0.82	4.51	7.29	54.66	105.42	1.18	401.12	0.85	601.95	0.25	69.48
RSD	< 20%			9.59%				10.93%							
Accuracy	± 30%			88.80%				106.92%							
HR_{PIR}				1.79											
Upper PIR	≤ 150%			125%											
Lower PIR	≥ 50%			53%											

**Assay Name:**	**SRP PFAS**	**New Method Validation**	
**Instrument:**	**Exploris 240**	**Regression:** **Linear**	**Weighting:** **1/x^2**
**Analyte:**	**PFPeS**		
**Internal Standard**	**PFHxA**		

DAY 1

**Calibrators (ng/L)**	~~1~~	**2**	**5**	**10**	**50**	**100**	**500**	**1,000**	**5,000**	~~10,000~~		

Date 10/27/2021

R^2 0.9935 1

**MRL =**	**2**	**ng/L**	**IDP =**	**3.41%**	**@ 500 ng/L**
**DL =**	**0.32**	**ng/L**	**IDA =**	**110.28%**	**@ 500 ng/L**

															**CON**
Rep		**1**	**2**	**5**	**10**	**50**	**100**	**500**	**1,000**	**Neg**	**5,000**	**Neg**	**10,000**	**Neg**	**800**
1			1.88	5.08	10.00	50.32	70.17	539.17	877.39	0.15	3,599.03	1.42		0.36	821.62
2			2.08	4.25	9.95	43.82	73.02	542.08	1,062.07	0.16	4,532.83	0.14		0.35	823.88
3			2.01	4.44	9.67	53.53	83.02	530.91	1,069.63	0.93	4,937.05	0.36		0.52	841.46
4			2.01	4.40	10.45	50.83	98.57	572.66	1,072.42	4.72	4,759.69	0.15		0.36	819.87
5			1.91	4.50	9.27	49.59	93.08	527.37	1,023.37	0.12	4,537.21	0.26		0.42	819.97
6			2.04	5.18	10.18	53.49	103.91	564.86	959.58	0.12	4,873.76	0.31		0.44	787.47
7			2.22	5.16	9.86	56.96	100.57	545.55	1,072.49	0.15	4,721.40	0.23		0.43	862.19
8			2.06	4.75	10.12	55.83	98.63	573.62	1,079.33	0.40	5,063.99	0.67		0.49	709.94
9			2.09	5.35	10.42	51.06	103.96	566.25	977.20	0.12	4,387.21	0.17		0.32	754.31
	N=		9	9	9	9	9	9	9	9	9	9		9	9
	t value		3.355	3.355	3.355	3.355	3.355	3.355	3.355	3.355	3.355	3.355		3.355	3.355
**Metric**	**Criteria**														
Mean			2.03	4.79	9.99	51.71	91.66	551.39	1021.50	0.76	4601.35	0.41		0.41	804.52
Std Dev			0.09	0.39	0.35	3.67	12.31	17.05	65.88	1.42	408.21	0.39		0.06	44.21
RSD	< 20%		4.74%					3.41%							
Accuracy	± 30%		101.67%					110.28%							
HR_{PIR}			0.35												
Upper PIR	≤ 150%		119%												
Lower PIR	≥ 50%		84%												

**Assay Name:**	**SRP PFAS**	**New Method Validation**		
**Instrument:**	**Exploris 240**	**Regression:**	**Linear**	**Weighting:** $1/x^2$
**Analyte:**	**PFHxS**			

DAY 1

**Calibrators (ng/L)**	~~1~~	~~2~~	~~5~~	**10**	**50**	**100**	**500**	**1,000**	**5,000**	**10,000**		
**Date**	**10/27/2021**											
**R^2**	**0.9933**	**4**										

**MRL =**	**10**	**ng/L**	**IDP =**	**7.86%**	**@ 500 ng/L**
**DL =**	**2.93**	**ng/L**	**IDA =**	**122.19%**	**@ 500 ng/L**

Rep		1	2	5	10	50	100	500	1,000	Neg	5,000	Neg	10,000	Neg	CON 800
1					9.93	54.65	100.60	639.20	1,243.80	0.33	4,938.01	0.81	8,251.85	1.68	943.13
2					9.21	52.36	81.38	604.10	948.94	(0.37)	4,503.75	3.38	8,175.63	1.15	916.99
3					9.71	43.91	81.78	558.23	1,108.12	0.05	5,113.23	1.10	8,781.76	1.30	859.75
4					8.82	41.19	74.70	646.61	1,185.26	6.80	5,547.78	0.30	8,471.39	0.56	896.26
5					11.41	48.95	96.58	643.94	1,149.21	0.05	4,575.62	0.43	8,667.60	0.24	812.63
6					10.01	58.96	110.19	650.18	1,211.25	(0.55)	5,232.15	(0.08)	11,297.64	0.41	874.92
7					9.77	41.85	76.75	533.18	1,082.75	(0.10)	6,058.78	(0.77)	10,760.77	(0.31)	711.39
8					11.04	55.28	110.14	624.20	1,027.92	0.22	5,938.96	0.39	11,264.04	1.39	940.45
9					11.34	56.67	97.83	598.79	958.23	0.87	4,963.11	1.29	8,778.29	1.09	829.74
	N=				9	9	9	9	9	9	9	9	9	9	9
	t value				3.355	3.355	3.355	3.355	3.355	3.355	3.355	3.355	3.355	3.355	3.355
**Metric**	**Criteria**														
Mean					10.14	50.42	92.22	610.94	1101.72	0.81	5207.93	0.76	9383.22	0.83	865.03
Std Dev					0.87	6.33	13.07	39.29	100.66	2.15	517.67	1.09	1243.19	0.61	69.27
RSD	< 20%				8.73%			7.86%							
Accuracy	± 30%				101.38%			122.19%							
HR_{PIR}					3.25										
Upper PIR	≤ 150%				134%										
Lower PIR	≥ 50%				69%										

**Assay Name:**	**SRP PFAS**	**New Method Validation**			
**Instrument:**	**Exploris 240**	**Regression:**	**Linear**	**Weighting:**	**$1/x^2$**
**Analyte:**	**PFHpS**				
**Internal Standard**	**6:2 FTS**				

DAY 1

**Calibrators (ng/L)**	~~**1**~~	**2**	**5**	**10**	**50**	**100**	**500**	**1,000**	**5,000**	**10,000**		

Date 10/27/2021

R^2 0.9986 1

**MRL =**	**2**	**ng/L**	**IDP =**	**12.74%**	**@ 500 ng/L**
**DL =**	**0.70**	**ng/L**	**IDA =**	**117.85%**	**@ 500 ng/L**

Rep		**1**	**2**	**5**	**10**	**50**	**100**	**500**	**1,000**	**Neg**	**5,000**	**Neg**	**10,000**	**Neg**	**CON 800**
1			1.89	5.71	10.03	55.81	59.37	560.36	950.64	-	5,944.78	5.31	11,330.41	1.37	808.30
2			2.06	6.00	11.04	69.68	58.79	552.65	1,027.90	0.03	4,817.04	0.59	9,184.08	2.15	867.66
3			1.84	5.09	9.52	60.90	82.63	555.72	1,320.50	3.14	5,957.03	0.58	11,468.07	1.80	1,111.38
4			*2.62	6.30	10.79	56.20	97.83	690.13	1,063.11	12.79	4,838.53	0.64	9,645.82	0.71	844.03
5			1.47	4.58	9.20	51.16	71.18	516.96	1,320.79	(0.16)	5,187.11	0.76	10,420.52	1.61	828.94
6			2.07	6.30	11.34	54.33	104.24	609.49	959.01	(0.16)	4,784.82	0.89	8,333.78	1.36	772.49
7			1.96	5.29	10.21	67.42	100.51	576.65	1,378.14	0.06	4,577.69	0.93	9,488.88	1.57	1,103.92
8			*2.46	4.40	10.78	66.47	87.35	708.03	1,019.17	0.88	4,607.30	2.26	8,122.12	1.74	787.76
9			1.84	5.61	10.08	56.63	75.85	533.44	1,031.00	0.03	4,814.43	0.58	8,264.00	2.52	830.21
	N=		7	9	9	9	9	9	9	9	9	9	9	9	9
	t value		3.707	3.355	3.355	3.355	3.355	3.355	3.355	3.355	3.355	3.355	3.355	3.355	3.355
**Metric**	**Criteria**														
Mean			1.88	5.48	10.33	59.84	81.97	589.27	1118.92	1.85	5058.75	1.39	9584.19	1.65	883.85
Std Dev			0.19	0.65	0.67	6.19	16.06	63.68	160.40	3.99	503.91	1.47	1197.90	0.48	122.60
RSD	< 20%		9.38%					12.74%							
Accuracy	± 30%		93.79%					117.85%							
HR_{PIR}			0.79												
Upper PIR	≤ 150%		134%												
Lower PIR	≥ 50%		54%												

Assay Name:	SRP PFAS	New Method Validation			
Instrument:	Exploris 240	Regression:	Linear	Weighting:	$1/x^2$
Analyte:	PFOS				

DAY 1

Calibrators (ng/L)		~~1~~	~~2~~	~~5~~	~~10~~	50	100	500	1,000	5,000	10,000		
Date	10/27/2021												
R^2	0.9963	1											

MRL =	50	ng/L	IDP =	9.81%	@ 500 ng/L
DL =	7.79	ng/L	IDA =	111.92%	@ 500 ng/L

Rep		1	2	5	10	50	100	500	1,000	Neg	5,000	Neg	10,000	Neg	CON 800
1						49.51	98.58	601.94	1,145.67	13.16	6,043.98	20.99	10,236.93	7.98	798.15
2						*74.98	91.61	612.89	944.86	6.71	5,713.13	6.22	9,515.47	8.91	733.34
3						50.99	76.63	506.57	1,137.54	20.33	5,629.54	18.75	9,776.63	20.43	909.19
4						55.64	108.52	533.13	940.17	17.91	4,598.80	9.64	12,552.83	7.02	770.92
5						49.53	83.35	526.27	953.63	9.51	5,812.99	14.53	11,499.25	20.08	747.27
6						51.07	102.76	516.56	949.06	7.25	4,638.64	17.00	8,840.30	12.55	748.85
7						*67.37	94.02	581.67	972.91	7.03	4,590.02	15.49	9,807.18	14.48	993.54
8						51.85	92.03	510.34	937.43	6.79	4,648.20	9.45	10,085.63	5.07	988.29
9						53.93	92.02	646.97	1,129.79	12.51	5,842.11	6.52	9,881.50	8.56	935.16
	N=					7	9	9	9	9	9	9	9	9	9
	t value					3.707	3.355	3.355	3.355	3.355	3.355	3.355	3.355	3.355	3.355
**Metric**	**Criteria**														
Mean						51.79	93.28	559.59	1012.34	11.24	5279.71	13.18	10243.97	11.68	847.19
Std Dev						2.10	9.02	49.05	89.21	4.82	600.38	5.09	1052.68	5.29	102.06
RSD	< 20%					4.20%		9.81%							
Accuracy	± 30%					103.58%		111.92%							
HR_{PIR}						8.90									
Upper PIR	≤ 150%					121%									
Lower PIR	≥ 50%					86%									

**Assay Name:**	**SRP PFAS**	**New Method Validation**		
**Instrument:**	**Exploris 240**	**Regression:** Linear	**Weighting:**	$1/x^2$
**Analyte:**	**PFNS**			
**Internal Standard**	**PFDA**			

DAY 1

**Calibrators (ng/L)**	~~1~~	2	5	10	50	100	500	1,000	5,000	~~10,000~~		

Date 10/27/2021

R^2 0.9992 1

**MRL =**	**2**	**ng/L**	**IDP =**	**2.65%**	**@ 500 ng/L**
**DL =**	**0.48**	**ng/L**	**IDA =**	**104.26%**	**@ 500 ng/L**

Rep		1	2	5	10	50	100	500	1,000	Neg	5,000	Neg	10,000	Neg	CON 800
1			2.3	5.02	9.79	49.08	77.18	533.42	986.52	0.92	4,564.75	8.49	8,117.06	3.31	810.00
2			2.23	5.07	10.09	53.75	75.00	527.99	1,044.05	0.89	4,791.50	2.55	8,286.91	5.32	830.45
3			2.2	4.86	10.59	50.14	80.12	518.49	1,002.02	2.97	4,677.24	2.11	8,598.52	4.07	808.08
4			2.21	5.13	10.23	50.09	88.57	501.81	983.75	10.75	4,842.89	2.56	7,833.94	3.47	778.14
5			2	4.94	8.81	47.81	83.45	506.24	940.81	0.59	4,645.07	2.10	8,394.53	3.70	771.00
6			2.3	5.13	9.90	50.76	96.25	528.75	947.85	0.56	4,730.53	2.45	8,022.55	2.99	721.28
7			1.98	5.28	9.65	50.45	89.73	505.73	965.28	0.64	4,627.20	2.72	8,236.66	3.05	794.09
8			1.88	4.96	10.36	50.98	95.66	527.29	975.82	2.45	4,749.87	2.61	8,376.47	4.44	788.33
9			2.12	5.09	9.94	48.84	98.45	542.15	1,000.45	0.20	4,807.63	1.98	8,661.53	3.59	797.03
	N=		9	9	9	9	9	9	9	9	9	9	9	9	9
	t value		3.355	3.355	3.355	3.355	3.355	3.355	3.355	3.355	3.355	3.355	3.355	3.355	3.355
**Metric**	**Criteria**														
Mean			2.14	5.05	9.93	50.21	87.16	521.32	982.95	2.22	4715.19	3.06	8280.91	3.77	788.71
Std Dev			0.14	0.12	0.48	1.58	8.18	13.23	29.42	3.14	87.46	1.93	250.15	0.70	29.14
RSD	< 20%		7.09%					2.65%							
Accuracy	± 30%		106.78%					104.26%							
HR_{PIR}			0.53												
Upper PIR	≤ 150%		133%												
Lower PIR	≥ 50%		80%												

**Assay Name:**	**SRP PFAS**	**New Method Validation**					
**Instrument:**	**Exploris 240**	**Regression:**	**Linear**	**Weighting:**	**$1/x^2$**		
**Analyte:**	**PFDS**						
**Internal Standard**	**8:2 FTS**						

DAY 1

**Calibrators (ng/L)**	**~~1~~**	**~~2~~**	**5**	**10**	**50**	**100**	**500**	**1,000**	**5,000**	**10,000**		

Date **10/27/2021**

R^2 **0.9979** **1**

**MRL =**	**5**	**ng/L**	**IDP =**	**9.12%**	**@ 500 ng/L**
**DL =**	**1.86**	**ng/L**	**IDA =**	**85.17%**	**@ 500 ng/L**

Rep		**1**	**2**	**5**	**10**	**50**	**100**	**500**	**1,000**	**Neg**	**5,000**	**Neg**	**10,000**	**Neg**	**CON 800**
1				4.6	11.49	48.09	78.75	*619.51	917.86	1.73	5,020.59	9.86	11,555.27	4.24	940.96
2				5.69	12.42	50.69	79.10	*620	1,209.35	1.73	6,828.01	3.63	9,285.10	5.17	977.23
3				4.68	8.94	51.72	96.84	389.89	942.80	3.67	4,028.45	1.93	8,976.97	3.61	766.10
4				5.34	10.27	37.22	81.11	486.74	1,055.31	5.23	5,519.77	2.98	8,494.57	3.78	693.14
5				4.97	9.99	45.33	103.14	366.18	953.12	1.73	6,474.79	2.40	11,440.37	4.69	929.68
6				4.71	9.04	50.91	85.45	456.74	862.02	1.73	6,150.47	2.33	8,952.68	3.07	562.96
7				*6.23	8.89	46.21	83.08	389.39	1,022.70	2.05	5,962.31	3.79	7,498.17	2.63	718.56
8				4.88	9.2	53.31	90.30	466.25	1,010.28	2.01	4,912.10	3.46	9,563.26	5.27	946.78
9				6.2	11.11	43.68	111.24	*616.66	1,181.21	1.78	6,303.29	1.73	11,409.28	4.29	957.56
	N=			8	9	9	9	6	9	9	9	9	9	9	9
	t value			3.499	3.355	3.355	3.355	4.032	3.355	3.355	3.355	3.355	3.355	3.355	3.355
**Metric**	**Criteria**														
Mean				5.13	10.15	47.46	89.89	425.87	1017.18	2.41	5688.86	3.57	9686.19	4.08	832.55
Std Dev				0.53	1.21	4.72	10.87	45.60	110.02	1.16	844.34	2.33	1372.91	0.85	141.55
RSD	< 20%			10.61%				9.12%							
Accuracy	± 30%			102.68%				85.17%							
HR_{PIR}				2.09											
Upper PIR	≤ 150%			144%											
Lower PIR	≥ 50%			61%											

Assay Name:	SRP PFAS	New Method Validation			
Instrument:	Exploris 240	Regression:	Linear	Weighting:	$1/x^2$
Analyte:	PFBA				

DAY 1

Calibrators (ng/L)	~~1~~	~~2~~	~~5~~	~~10~~	50	100	500	1,000	5,000	10,000		

Date 10/27/2021

R^2 0.9906 3

MRL =	50	ng/L	IDP =	1.08%	@ 500 ng/L
DL =	19.08	ng/L	IDA =	113.30%	@ 500 ng/L

Rep		1	2	5	10	50	100	500	1,000	Neg	5,000	Neg	10,000	Neg	CON 800
1						49.45	94.12	576.24	1,077.99	13.06	5,326.12	8.56	9,378.45	22.37	825.59
2						48.62	78.79	558.93	1,046.46	8.31	5,004.78	16.28	8,902.30	16.28	820.42
3						58.35	79.99	565.00	1,067.12	6.24	5,480.30	19.02	10,120.17	25.67	818.67
4						47.46	93.54	566.37	1,095.84	2.70	5,152.80	14.84	9,429.88	8.32	842.60
5						49.00	93.09	565.42	1,045.46	9.01	5,060.31	14.02	9,518.93	5.59	824.78
6						53.17	95.44	563.39	1,077.29	(3.79)	5,211.14	1.64	10,108.36	5.89	821.79
7						61.57	100.41	570.42	1,053.03	1.60	5,231.38	17.96	10,154.78	25.19	818.28
8						62.48	97.77	572.76	1,098.91	11.47	5,288.70	15.79	9,568.30	24.45	809.62
9						48.43	105.74	559.82	1,063.84	(3.16)	5,078.77	16.46	9,557.75	28.29	822.33
	N=					9	9	9	9	9	9	9	9	9	9
	t value					3.355	3.355	3.355	3.355	3.355	3.355	3.355	3.355	3.355	3.355
**Metric**	**Criteria**														
Mean						53.17	93.21	566.48	1069.55	5.05	5203.81	13.84	9637.66	18.01	822.68
Std Dev						5.69	8.29	5.42	18.59	5.74	140.58	5.14	394.15	8.66	8.31
RSD	< 20%					11.37%		1.08%							
Accuracy	± 30%					106.34%		113.30%							
HR_{PIR}						21.20									
Upper PIR	≤ 150%					149%									
Lower PIR	≥ 50%					64%									

Assay Name:	SRP PFAS	New Method Validation			
Instrument:	Exploris 240	Regression:	Linear	Weighting:	$1/x^2$
Analyte:	PFPeA				

DAY 1

Calibrators (ng/L)	~~1~~	~~2~~	~~5~~	10	50	100	500	1,000	5,000	10,000		

Date 10/27/2021

R^2 0.9881 1

MRL =	10	ng/L	IDP =	7.09%	@ 500 ng/L
DL =	3.91	ng/L	IDA =	104.00%	@ 500 ng/L

Rep		1	2	5	10	50	100	500	1,000	Neg	5,000	Neg	10,000	Neg	CON 800
1					10.97	50.68	78.33	477.68	922.09	1.85	4,184.31	2.26	8,300.01	5.82	815.14
2					7.36	44.97	75.50	488.62	1,037.05	1.76	5,158.25	3.71	8,406.79	6.92	738.87
3					9.03	53.65	79.50	561.48	943.67	2.02	4,033.75	2.29	7,501.22	1.65	646.66
4					9.63	45.26	83.18	533.86	1,088.80	2.97	5,269.53	2.42	8,537.03	2.30	770.85
5					9.73	53.57	91.75	562.50	1,077.07	0.34	4,944.51	1.34	9,574.29	0.81	764.05
6					8.85	50.38	92.54	550.31	1,059.64	0.32	4,860.39	1.48	8,964.72	1.61	779.77
7					9.59	48.22	80.83	466.96	1,010.23	0.11	5,102.89	2.36	8,106.41	3.87	686.64
8					11.33	53.47	101.22	542.55	1,119.81	1.22	4,617.94	2.51	9,387.43	4.08	780.94
9					10.84	52.63	93.91	495.85	927.13	(0.40)	5,002.59	2.73	9,667.79	4.77	707.45
	N=				9	9	9	9	9	9	9	9	9	9	9
	t value				3.355	3.355	3.355	3.355	3.355	3.355	3.355	3.355	3.355	3.355	3.355
**Metric**	**Criteria**														
Mean					9.70	50.31	86.31	519.98	1020.61	1.13	4797.13	2.34	8716.19	3.54	743.37
Std Dev					1.17	3.26	8.27	35.46	69.88	1.04	408.98	0.65	691.47	1.97	50.50
RSD	< 20%				11.66%			7.09%							
Accuracy	± 30%				97.03%			104.00%							
HR_{PIR}					4.35										
Upper PIR	≤ 150%				141%										
Lower PIR	≥ 50%				54%										

Assay Name:	SRP PFAS	New Method Validation			
Instrument:	Exploris 240	Regression:	Linear	Weighting:	$1/x^2$
Analyte:	PFHxA				

DAY 1

Calibrators (ng/L)	~~1~~	~~2~~	5	10	50	100	500	1,000	5,000	~~10,000~~		

Date	10/27/2021	
R^2	0.9835	1

MRL =	5	ng/L	IDP =	8.34%	@ 500 ng/L
DL =	1.74	ng/L	IDA =	117.01%	@ 500 ng/L

Rep		1	2	5	10	50	100	500	1,000	Neg	5,000	Neg	10,000	Neg	CON 800
1				4.79	10.18	49.44	79.71	602.62	1,109.84	(0.03)	5,363.67	0.64	9,041.31	1.17	796.85
2				5.63	10.46	48.96	72.36	522.72	1,028.56	(0.04)	4,646.45	0.43	9,103.10	1.19	797.65
3				5.02	9.56	52.61	79.63	620.91	1,306.30	2.27	6,427.65	1.89	11,632.22	1.45	1,020.46
4				5.91	11.2	61.6	113.56	642.43	1,057.39	4.03	4,848.63	0.64	9,513.98	0.63	867.71
5				4.64	10.17	48.92	88.09	570.92	1,159.72	0.61	4,984.19	0.07	8,693.09	(0.05)	782.88
6				*6.7	12.72	60.09	121.43	*669.78	1,116.13	(0.57)	4,973.67	1.89	9,887.98	1.62	849.41
7				*6.81	12.1	64.97	114.24	*701.85	1,236.38	(0.83)	5,005.01	1.27	10,388.61	1.38	1,029.63
8				5.42	10.39	56.63	94.15	603.99	1,079.57	0.31	5,682.48	1.37	9,469.63	0.52	805.87
9				5.83	9.67	47.8	88.94	531.86	1,064.51	(0.78)	5,331.24	0.08	9,356.08	0.83	819.79
	N=			7	9	9	9	7	9	9	9	9	9	9	9
	t value			3.707	3.355	3.355	3.355	3.707	3.355	3.355	3.355	3.355	3.355	3.355	3.355
**Metric**	**Criteria**														
Mean				5.32	10.72	54.56	94.68	585.06	1128.71	0.55	5251.44	0.92	9676.22	0.97	863.36
Std Dev				0.47	1.02	6.07	16.60	41.68	85.80	1.52	508.35	0.67	833.19	0.50	90.04
RSD	< 20%			9.40%				8.34%							
Accuracy	± 30%			106.40%				117.01%							
HR_{PIR}				1.99											
Upper PIR	≤ 150%			146%											
Lower PIR	≥ 50%			67%											

**Assay Name:**	**SRP PFAS**	**New Method Validation**	
**Instrument:**	**Exploris 240**	**Regression:** Linear	**Weighting:** $1/x^2$
**Analyte:**	**PFHpA**		

DAY 1

**Calibrators (ng/L)**	~~1~~	~~2~~	5	10	50	100	500	1,000	5,000	10,000		

Date 10/27/2021

R^2 0.9968 1

**MRL =**	**5**	**ng/L**	**IDP =**	**6.80%**	**@ 500 ng/L**
**DL =**	**1.56**	**ng/L**	**IDA =**	**111.06%**	**@ 500 ng/L**

Rep		**1**	**2**	**5**	**10**	**50**	**100**	**500**	**1,000**	**Neg**	**5,000**	**Neg**	**10,000**	**Neg**	**CON 800**
1				6.01	10.38	48.38	86.88	556.02	1,134.16	0.85	5,466.11	2.60	9,668.37	1.92	791.41
2				*6.58	11.08	56.45	75.33	514.41	1,017.48	0.57	4,674.05	0.97	9,105.22	1.95	739.52
3				5.02	10.68	50.59	77.28	618.36	1,411.25	3.41	6,525.94	2.31	11,324.70	3.69	*1111.21
4				5.99	14.28	59.06	115.80	568.29	1,076.75	8.25	4,700.04	1.16	9,773.81	1.45	913.08
5				5.03	9.34	47.23	83.40	572.65	1,163.65	1.72	5,420.77	0.89	10,257.87	1.25	754.98
6				*8.47	12.94	50.56	113.19	511.96	942.15	0.67	4,886.52	2.56	9,624.74	3.36	942.12
7				5.56	13.66	66.80	115.71	*705.82	1,361.49	1.07	4,887.41	2.44	9,659.77	3.49	1,048.99
8				5.87	10.05	56.36	94.27	*635.94	1,053.15	2.26	5,003.67	2.98	9,391.79	1.60	820.27
9				6.04	10.28	46.70	97.33	545.43	1,074.16	0.75	5,190.71	1.37	10,009.78	1.70	756.66
	N=			7	9	9	9	7	9	9	9	9	9	9	8
	t value			3.707	3.355	3.355	3.355	3.707	3.355	3.355	3.355	3.355	3.355	3.355	3.499
**Metric**	**Criteria**														
Mean				5.65	11.41	53.57	95.47	555.30	1137.14	2.17	5195.02	1.92	9868.45	2.27	845.88
Std Dev				0.42	1.66	6.27	15.27	34.01	146.51	2.32	541.46	0.76	601.29	0.91	103.80
RSD	< 20%			8.40%				6.80%							
Accuracy	± 30%			112.91%				111.06%							
HR_{PIR}				1.78											
Upper PIR	≤ 150%			149%											
Lower PIR	≥ 50%			77%											

Assay Name:	SRP PFAS	New Method Validation			
Instrument:	Exploris 240	Regression:	Linear	Weighting:	$1/x^2$
Analyte:	PFOA				

DAY 1

Calibrators (ng/L)	~~1~~	~~2~~	~~5~~	10	50	100	500	1,000	5,000	10,000		

Date 10/27/2021

R^2 0.9941 9

MRL =	10	ng/L	IDP =	3.57%	@ 500 ng/L
DL =	4.08	ng/L	IDA =	109.96%	@ 500 ng/L

Rep		1	2	5	10	50	100	500	1,000	Neg	5,000	Neg	10,000	Neg	CON 800
1					*5.71	34.64	88.42	561.54	1,028.60	1.31	4,964.22	2.23	9,116.88	(0.26)	802.96
2					10.72	52.34	72.45	508.49	793.31	(5.94)	3,603.88	1.65	7,108.85	(1.33)	798.37
3					*5.73	40.48	57.31	547.47	1,038.09	(0.90)	4,775.84	(3.41)	6,986.36	(0.02)	797.80
4					*5.57	50.55	76.26	541.73	1,048.21	4.24	4,965.58	2.88	9,209.20	2.41	817.02
5					9.74	51.03	89.06	548.26	1,049.20	10.18	4,858.68	(0.61)	6,993.40	2.66	807.39
6					11.03	49.89	84.31	563.92	1,015.14	1.03	4,985.51	(1.67)	8,048.23	1.98	806.53
7					8.59	51.38	97.06	559.93	1,038.18	1.44	5,051.35	1.25	9,339.88	2.24	801.87
8					10.42	49.51	89.65	574.24	1,038.95	(0.86)	4,941.45	(1.15)	9,510.31	2.83	809.52
9					8.41	49.35	96.46	542.71	1,035.66	(1.38)	4,601.24	0.73	8,827.52	3.78	717.95
	N=				6	9	9	9	9	9	9	9	9	9	9
	t value				4.032	3.355	3.355	3.355	3.355	3.355	3.355	3.355	3.355	3.355	3.355
**Metric**	**Criteria**														
Mean					9.82	47.69	83.44	549.81	1009.48	1.01	4749.75	0.21	8348.96	1.59	795.49
Std Dev					1.01	5.65	12.05	17.85	77.03	4.16	424.72	1.94	1011.70	1.61	27.98
RSD	< 20%				10.12%			3.57%							
Accuracy	± 30%				98.18%			109.96%							
HR_{PIR}					4.76										
Upper PIR	≤ 150%				146%										
Lower PIR	≥ 50%				51%										

**Assay Name:**	**SRP PFAS**	**New Method Validation**			
**Instrument:**	**Exploris 240**	**Regression:**	**Linear**	**Weighting:**	**$1/x^2$**
**Analyte:**	**PFNA**				

DAY 1

**Calibrators (ng/L)**	~~1~~	~~2~~	**5**	**10**	**50**	**100**	**500**	**1,000**	**5,000**	~~10,000~~		

Date 10/27/2021

R^2 0.9932 1

**MRL =**	**5**	**ng/L**	**IDP =**	**1.45%**	**@ 500 ng/L**
**DL =**	**1.60**	**ng/L**	**IDA =**	**111.32%**	**@ 500 ng/L**

Rep		**1**	**2**	**5**	**10**	**50**	**100**	**500**	**1,000**	**Neg**	**5,000**	**Neg**	**10,000**	**Neg**	**CON 800**
1				5.67	10.44	47.34	81.60	557.63	1,039.81	0.77	5,037.56	3.44	8,899.42	4.21	837.71
2				4.28	10.66	51.67	76.23	553.31	1,043.02	0.89	4,918.23	2.91	9,048.50	2.15	817.81
3				4.85	9.67	53.37	75.76	549.60	1,038.30	3.98	5,010.86	3.94	9,313.85	3.25	815.89
4				5.16	10.51	51.52	88.45	554.44	1,040.41	11.28	4,992.65	2.27	9,239.98	3.09	803.59
5				*7.18	11.47	51.58	85.40	560.33	1,007.29	1.29	5,083.62	2.10	9,398.57	3.80	824.21
6				5.82	11.08	51.37	98.78	555.10	1,045.86	1.09	4,998.31	2.77	9,200.42	3.65	811.77
7				5.28	10.45	51.46	90.01	571.03	1,050.51	0.40	4,963.99	2.09	9,512.54	3.16	824.95
8				4.97	10.37	51.67	101.21	563.04	1,066.77	1.82	4,983.91	1.66	9,421.04	3.89	816.63
9				5.42	11.07	49.30	93.86	544.82	1,031.83	0.89	4,913.75	2.64	9,416.09	2.47	806.02
	N=			8	9	9	9	9	9	9	9	9	9	9	9
	t value			3.499	3.355	3.355	3.355	3.355	3.355	3.355	3.355	3.355	3.355	3.355	3.355
**Metric**	**Criteria**														
Mean				5.18	10.64	51.03	87.92	556.59	1040.42	2.49	4989.21	2.65	9272.27	3.30	817.62
Std Dev				0.46	0.49	1.62	8.60	7.23	14.94	3.26	50.78	0.68	186.64	0.63	9.83
RSD	< 20%			9.17%				1.45%							
Accuracy	± 30%			103.63%				111.32%							
HR_{PIR}				1.80											
Upper PIR	≤ 150%			140%											
Lower PIR	≥ 50%			68%											

Assay Name:	SRP PFAS	New Method Validation			
Instrument:	Exploris 240	Regression:	Linear	Weighting:	$1/x^2$
Analyte:	PFDA				

DAY 1

Calibrators (ng/L)	~~1~~	2	5	10	50	100	500	1,000	5,000	10,000		

Date	10/27/2021	
R^2	0.9952	1

MRL =	2	ng/L	IDP =	0.97%	@ 500 ng/L
DL =	0.53	ng/L	IDA =	110.39%	@ 500 ng/L

Rep		1	2	5	10	50	100	500	1,000	Neg	5,000	Neg	10,000	Neg	CON 800
1			*2.63	5.19	9.37	51.18	78.85	548.04	987.54	1.20	4,168.35	5.80	9,218.42	5.08	830.58
2			2.33	4.94	10.54	48.44	77.74	548.35	1,052.48	1.23	5,022.40	2.91	9,671.80	4.95	817.31
3			2.2	4.85	8.59	53.37	75.99	547.48	1,036.54	3.28	5,211.09	3.10	9,735.56	4.48	825.53
4			*1.98	4.51	9.62	52.73	89.97	545.00	1,040.01	9.53	5,226.01	2.69	9,404.97	4.24	812.47
5			2.41	4.70	9.56	51.73	85.14	556.47	992.52	1.59	5,097.16	2.74	9,415.68	4.34	814.83
6			2.49	4.88	10.38	51.66	97.49	555.91	1,032.70	0.82	5,145.99	3.18	9,707.14	4.61	816.55
7			2.08	4.50	10.99	52.56	91.39	559.76	1,035.96	0.95	5,147.51	3.64	9,800.79	3.92	822.58
8			2.43	5.08	10.36	51.79	97.07	550.67	1,031.30	2.24	5,248.97	3.57	9,700.87	5.20	818.47
9			2.49	5.38	9.64	51.07	97.47	555.88	1,024.52	1.18	5,193.09	2.75	9,758.45	4.55	803.13
	N=		7	9	9	9	9	9	9	9	9	9	9	9	9
	t value		3.707	3.355	3.355	3.355	3.355	3.355	3.355	3.355	3.355	3.355	3.355	3.355	3.355
**Metric**	**Criteria**														
Mean			2.35	4.89	9.89	51.61	87.90	551.95	1025.95	2.45	5051.17	3.38	9601.52	4.60	817.94
Std Dev			0.14	0.28	0.69	1.33	8.29	4.84	20.50	2.61	319.03	0.92	190.96	0.39	7.46
RSD	< 20%		7.19%					0.97%							
Accuracy	± 30%		117.36%					110.39%							
HR_{PIR}			0.61												
Upper PIR	≤ 150%		148%												
Lower PIR	≥ 50%		87%												

**Assay Name:**	**SRP PFAS**	**New Method Validation**			
**Instrument:**	**Exploris 240**	**Regression:**	**Linear**	**Weighting:**	**$1/x^2$**
**Analyte:**	**PFUdA**				

DAY 1

**Calibrators (ng/L)**	~~**1**~~	~~**2**~~	~~**5**~~	**10**	**50**	**100**	**500**	**1,000**	**5,000**	**10,000**		

Date 10/27/2021

R^2 0.9992 10

**MRL =**	**5**	**ng/L**	**IDP =**	**8.84%**	**@ 500 ng/L**
**DL =**	**1.88**	**ng/L**	**IDA =**	**118.56%**	**@ 500 ng/L**

															**CON**
Rep		**1**	**2**	**5**	**10**	**50**	**100**	**500**	**1,000**	**Neg**	**5,000**	**Neg**	**10,000**	**Neg**	**800**
1				*3.37	8.13	49.72	89.95	595.17	1,106.17	0.45	5,352.40	2.99	8,246.02	5.65	865.26
2				5.34	8.83	45.85	82.53	480.35	893.73	(1.08)	5,260.80	6.35	8,039.85	5.07	843.51
3				*3.3	8.40	51.17	69.16	602.73	1,083.52	(1.02)	4,825.64	3.04	8,878.09	7.08	805.11
4				*3.51	7.77	54.57	76.24	584.03	1,228.45	1.29	5,344.95	3.12	10,668.05	5.65	944.60
5				5.16	10.82	54.78	103.29	611.53	1,119.16	5.91	4,960.94	1.88	9,970.31	5.18	954.97
6				4.20	9.69	52.69	85.60	575.25	1,149.82	(0.34)	4,852.13	1.79	9,186.31	5.51	865.08
7				*3.26	11.41	54.82	110.69	640.55	998.85	(0.42)	5,259.85	3.71	10,581.94	6.46	924.68
8				4.69	9.26	54.29	102.92	618.71	1,162.90	(0.37)	5,246.49	3.38	10,717.93	6.06	976.43
9				4.60	8.17	52.71	95.18	626.94	1,080.73	1.78	5,549.97	3.56	10,296.24	6.59	820.31
	N=			5	9	9	9	9	9	9	9	9	9	9	9
	t value			4.604	3.355	3.355	3.355	3.355	3.355	3.355	3.355	3.355	3.355	3.355	3.355
**Metric**	**Criteria**														
Mean				4.80	9.16	52.29	90.62	592.81	1091.48	0.69	5183.69	3.31	9620.53	5.92	888.88
Std Dev				0.41	1.19	2.82	12.88	44.22	91.96	2.06	233.83	1.25	996.24	0.64	58.99
RSD	< 20%			8.16%				8.84%							
Accuracy	± 30%			95.96%				118.56%							
HR_{PIR}				2.26											
Upper PIR	≤ 150%			141%											
Lower PIR	≥ 50%			51%											

**Assay Name:**	**SRP PFAS**	**New Method Validation**							
**Instrument:**	**Exploris 240**	**Regression:**	**Linear**	**Weighting:**	**$1/x^2$**				
**Analyte:**	**PFDoA**								

DAY 1

**Calibrators (ng/L)**	**~~1~~**	**~~2~~**	**~~5~~**	**10**	**50**	**100**	**500**	**1,000**	**5,000**	**10,000**		

Date **10/27/2021**

R^2 **0.9919** **1**

**MRL =**	**10**	**ng/L**	**IDP =**	**9.15%**	**@ 500 ng/L**
**DL =**	**3.41**	**ng/L**	**IDA =**	**105.40%**	**@ 500 ng/L**

Rep		**1**	**2**	**5**	**10**	**50**	**100**	**500**	**1,000**	**Neg**	**5,000**	**Neg**	**10,000**	**Neg**	**CON 800**
1					9.19	50.56	85.68	608.48	968.50	1.39	5,791.02	10.66	10,718.07	7.11	842.10
2					11.16	64.94	79.60	531.37	969.04	0.79	5,958.49	5.67	10,787.03	9.57	862.22
3					11.2	50.12	83.11	483.54	921.85	1.11	4,216.41	3.47	8,908.28	7.62	730.69
4					9.83	46.39	80.88	508.07	912.33	3.02	5,286.43	4.98	11,533.94	8.03	718.96
5					8.45	49.51	83.06	482.65	850.08	0.08	6,076.71	6.44	11,518.37	9.90	758.14
6					8.66	47.38	94.16	513.53	919.38	0.70	5,041.07	3.27	9,318.73	7.80	735.83
7					8.36	50.55	81.82	484.97	827.10	0.31	5,233.45	4.23	9,062.25	7.00	735.20
8					9.79	50.01	99.63	526.03	982.55	1.53	4,927.68	3.38	11,340.71	9.46	791.70
9					9	48.74	98.95	604.51	1,130.43	0.15	6,092.93	5.25	11,178.33	10.10	778.83
	N=				9	9	9	9	9	9	9	9	9	9	9
	t value				3.355	3.355	3.355	3.355	3.355	3.355	3.355	3.355	3.355	3.355	3.355
**Metric**	**Criteria**														
Mean					9.52	50.91	87.43	527.02	942.36	1.01	5402.69	5.26	10485.08	8.51	772.63
Std Dev					1.02	5.14	7.48	45.74	83.02	0.86	595.94	2.17	1021.82	1.17	48.14
RSD	< 20%				10.16%			9.15%							
Accuracy	± 30%				95.16%			105.40%							
HR_{PIR}					3.79										
Upper PIR	≤ 150%				133%										
Lower PIR	≥ 50%				57%										

**Assay Name:**	**SRP PFAS**	**New Method Validation**				
**Instrument:**	**Exploris 240**	**Regression:**	**Linear**	**Weighting:**	**$1/x^2$**	
**Analyte:**	**PFTrDA**					
**Internal Standard**	**PFDoA**					

DAY 1

**Calibrators (ng/L)**	**~~1~~**	**~~2~~**	**~~5~~**	**10**	**50**	**100**	**500**	**1,000**	**5,000**	**10,000**		

Date 10/27/2021

R^2 0.9722 1

**MRL =**	**10**	**ng/L**	**IDP =**	**8.59%**	**@ 500 ng/L**
**DL =**	**2.80**	**ng/L**	**IDA =**	**99.14%**	**@ 500 ng/L**

Rep		1	2	5	10	50	100	500	1,000	Neg	5,000	Neg	10,000	Neg	CON 800
1					8.28	40.45	82.14	584.89	980.20	1.55	5,908.89	12.78	11,907.85	11.98	906.24
2					11.31	54.64	80.52	496.42	1,008.39	2.63	6,250.78	7.42	11,817.94	13.67	883.02
3					9.71	46.65	76.84	455.32	1,020.40	4.43	4,697.69	5.75	10,401.37	11.29	850.73
4					10.05	43.73	79.53	478.20	987.29	3.39	5,000.99	5.52	12,997.60	11.20	732.38
5					9.05	45.93	77.20	431.62	862.79	1.96	6,265.88	6.97	12,319.00	12.86	758.42
6					8.96	46.53	98.32	499.81	940.11	2.24	5,010.43	6.32	11,166.93	11.13	712.75
7					9.07	46.65	78.93	486.98	910.25	1.80	5,355.85	6.09	10,168.24	10.04	715.17
8					10.05	48.25	91.81	483.67	952.05	1.93	5,168.86	6.18	13,771.85	15.07	795.00
9					9.08	46.03	95.53	544.47	1,161.79	1.45	6,151.90	6.74	13,124.34	12.91	782.39
	N=				9	9	9	9	9	9	9	9	9	9	9
	t value				3.355	3.355	3.355	3.355	3.355	3.355	3.355	3.355	3.355	3.355	3.355
**Metric**	**Criteria**														
Mean					9.51	46.54	84.54	495.71	980.36	2.38	5534.59	7.09	11963.90	12.24	792.90
Std Dev					0.83	3.56	7.85	42.97	79.24	0.92	576.82	2.09	1161.20	1.45	68.10
RSD	< 20%				8.35%			8.59%							
Accuracy	± 30%				95.07%			99.14%							
HR_{PIR}					3.11										
Upper PIR	≤ 150%				126%										
Lower PIR	≥ 50%				64%										

**Assay Name:**	**SRP PFAS**	**New Method Validation**	
**Instrument:**	**Exploris 240**	**Regression:** Linear	**Weighting:** $1/x^2$
**Analyte:**	**PFTeDA**		

DAY 1

**Calibrators (ng/L)**	~~1~~	~~2~~	~~5~~	10	50	100	500	1,000	5,000	10,000		

Date 10/27/2021

R^2 0.9885 1

**MRL =**	**10**	**ng/L**	**IDP =**	**12.52%**	**@ 500 ng/L**
**DL =**	**2.44**	**ng/L**	**IDA =**	**123.28%**	**@ 500 ng/L**

Rep		1	2	5	10	50	100	500	1,000	Neg	5,000	Neg	10,000	Neg	CON 800
1					11.05	55.64	97.52	524.45	989.59	2.59	6,520.35	11.80	10,283.47	14.77	1,003.43
2					*13.24	49.96	99.39	622.42	1,005.17	1.19	5,554.69	3.12	9,879.24	12.13	908.28
3					11.36	62.48	99.74	636.19	1,200.64	3.83	7,019.94	6.77	12,501.41	20.45	1,173.04
4					*13.13	52.34	102.28	538.86	1,090.52	2.91	5,088.18	6.06	12,900.39	18.68	947.68
5					*8.48	54.28	91.92	558.63	975.20	1.19	5,459.05	5.60	12,332.14	17.82	897.55
6					10.55	53.40	130.97	680.44	1,048.78	1.19	5,228.87	10.12	12,595.39	16.04	904.46
7					11.15	62.57	117.35	656.60	1,184.52	3.13	5,080.41	7.03	12,540.16	16.14	1,133.88
8					12.54	57.66	116.10	607.30	1,025.65	1.19	5,247.41	3.62	13,609.04	18.98	917.40
9					11.47	57.06	120.79	722.50	1,190.41	1.19	5,410.20	5.40	14,988.32	24.71	973.63
	N=				6	9	9	9	9	9	9	9	9	9	9
	t value				4.032	3.355	3.355	3.355	3.355	3.355	3.355	3.355	3.355	3.355	3.355
**Metric**	**Criteria**														
Mean					11.35	56.15	108.45	616.38	1078.94	2.05	5623.23	6.61	12403.28	17.75	984.37
Std Dev					0.61	4.06	12.41	62.62	85.93	1.00	642.08	2.65	1465.08	3.40	96.59
RSD	< 20%				6.06%			12.52%							
Accuracy	± 30%				113.53%			123.28%							
HR_{PIR}					2.85										
Upper PIR	≤ 150%				142%										
Lower PIR	≥ 50%				85%										

Assay Name:	SRP PFAS	New Method Validation			
Instrument:	Exploris 240	Regression:	Linear	Weighting:	$1/x^2$
Analyte:	PFHxDA				

DAY 1

Calibrators (ng/L)	~~1~~	~~2~~	~~5~~	~~10~~	~~50~~	100	500	1,000	5,000	10,000		

Date 10/27/2021

R^2 0.9279 6

MRL =	100	ng/L	IDP =	10.99%	@ 500 ng/L
DL =	32.37	ng/L	IDA =	96.89%	@ 500 ng/L

Rep		1	2	5	10	50	100	500	1,000	Neg	5,000	Neg	10,000	Neg	CON 800
1							106.52	541.89	1,357.17	72.38	7,573.42	86.42	9,582.98	159.74	822.76
2							117.7	519.3	1,081.41	72.38	6,565.07	72.38	10,565.37	141.60	711.77
3							94.74	534.06	838.54	72.38	5,281.29	76.22	9,695.66	129.70	647.31
4							108.29	*396.78	946.04	78.70	5,476.59	84.12	11,205.65	130.07	707.57
5							94.95	405.57	*641.27	72.38	6,348.75	72.38	13,121.89	122.21	613.35
6							120.32	*314.33	759.96	72.38	6,280.26	76.00	13,217.89	119.58	597.10
7							120.51	491.17	846.45	72.38	6,575.69	72.38	13,988.96	114.61	567.96
8							118.97	414.58	*726.81	72.38	5,185.37	72.38	*15511.27	148.63	671.52
9							107.44	*379.92	*701.24	72.38	6,039.80	72.38	*15282.59	117.22	714.11
	N=						9	6	6	9	9	9	7	9	9
	t value						3.355	4.032	4.032	3.355	3.355	3.355	3.707	3.355	3.355
**Metric**	**Criteria**														
Mean							109.94	484.43	971.60	73.08	6147.36	76.07	11625.49	131.48	672.61
Std Dev							9.65	54.96	199.81	1.99	713.90	5.16	1671.49	14.59	72.90
RSD	< 20%						9.65%	10.99%							
Accuracy	± 30%						109.94%	96.89%							
HR_{PIR}							35.97								
Upper PIR	≤ 150%						146%								
Lower PIR	≥ 50%						74%								

**Assay Name:**	**SRP PFAS**	**New Method Validation**	
**Instrument:**	**Exploris 240**	**Regression:** **Linear**	**Weighting:** **$1/x^2$**
**Analyte:**	**PFODA**		
**Internal Standard**	**PFTeDA**		

DAY 1

**Calibrators (ng/L)**	**~~1~~**	**~~2~~**	**~~5~~**	**~~10~~**	**~~50~~**	**~~100~~**	**500**	**1,000**	**5,000**	**10,000**		

Date **10/27/2021**

R^2 **0.9611** **1**

**MRL =**	**500**	**ng/L**	**IDP =**	**6.49%**	**@ 500 ng/L**
**DL =**	**120.26**	**ng/L**	**IDA =**	**78.36%**	**@ 500 ng/L**

Rep		**1**	**2**	**5**	**10**	**50**	**100**	**500**	**1,000**	**Neg**	**5,000**	**Neg**	**10,000**	**Neg**	**CON 800**
1								435.06	987.99	230.06	4,974.44	233.36	7,574.16	250.85	636.42
2								426.86	769.61	230.06	3,894.01	234.68	7,994.11	247.66	601.11
3								392.82	839.46	230.06	2,822.62	232.89	6,751.46	244.87	449.25
4								400.92	652.97	230.06	4,015.37	232.56	6,655.84	244.72	540.59
5								391.18	640.60	230.70	2,935.83	233.94	7,138.71	244.64	453.89
6								335.31	569.93	230.06	2,532.28	231.87	5,541.29	239.35	421.24
7								*329.57	510.77	230.06	2,236.14	230.06	6,042.98	241.61	393.40
8								360.35	550.18	230.06	2,645.92	230.48	5,618.24	242.04	399.37
9								*327.46	478.48	230.06	2,090.43	230.06	5,615.46	244.49	372.25
	N=							7	9	9	9	9	9	9	9
	t value							3.707	3.355	3.355	3.355	3.355	3.355	3.355	3.355
**Metric**	**Criteria**														
Mean								391.79	666.67	230.13	3127.45	232.21	6548.03	244.47	474.17
Std Dev								32.44	158.89	0.20	905.11	1.61	853.02	3.19	90.18
RSD	< 20%							6.49%	15.89%						
Accuracy	± 30%							78.36%	66.67%						
HR_{PIR}								137.44							
Upper PIR	≤ 150%							106%							
Lower PIR	≥ 50%							51%							

**Assay Name:**	**SRP PFAS**	**New Method Validation**			
**Instrument:**	**Exploris 240**	**Regression:**	**Linear**	**Weighting:**	**$1/x^2$**
**Analyte:**	**HFPO-DA (Gen-X) Decarboxylate**				

DAY 1

**Calibrators (ng/L)**	~~**1**~~	~~**2**~~	**5**	**10**	**50**	**100**	**500**	**1,000**	**5,000**	~~**10,000**~~		

Date **10/27/2021**

R^2 **0.9983** **1**

**MRL =**	**5**	**ng/L**	**IDP =**	**9.27%**	**@ 500 ng/L**
**DL =**	**1.14**	**ng/L**	**IDA =**	**105.81%**	**@ 500 ng/L**

															**CON**
Rep		**1**	**2**	**5**	**10**	**50**	**100**	**500**	**1,000**	**Neg**	**5,000**	**Neg**	**10,000**	**Neg**	**800**
1				4.08	10.78	48.19	82.76	609.30	1,144.14	0.08	5,543.45	0.43	9,704.62		882.50
2				*5.63	9.00	50.55	75.29	563.21	1,101.94	(1.22)	5,380.16	(0.16)	9,972.24	(0.40)	837.76
3				4.64	10.78	46.17	70.20	501.57	910.18	1.11	4,449.84	1.16	7,719.40	1.54	732.85
4				4.11	8.41	44.13	84.79	496.27	979.16	2.59	5,316.92	0.19	10,048.33	(0.48)	702.39
5				3.74	9.55	47.23	75.07	484.65	930.67	(1.31)	5,416.17	(0.76)	10,480.40	0.11	873.37
6				3.5	7.41	42.60	84.91	510.98	988.90	(2.58)	5,037.18	(1.31)	9,290.98	(0.27)	672.03
7				3.83	7.91	50.05	85.56	507.46	880.66	(1.03)	5,304.70	0.33	9,099.86	(0.89)	721.56
8				4.03	8.73	43.96	86.48	486.27	961.60	(0.24)	4,777.07	0.25	10,161.55	(1.25)	871.73
9				4.27	9.11	48.98	91.08	601.75	1,133.91	(0.10)	5,574.87	(1.00)	10,328.44	(0.73)	849.79
	N=			8	9	9	9	9	9	9	9	9	9	8	9
	t value			3.499	3.355	3.355	3.355	3.355	3.355	3.355	3.355	3.355	3.355	3.499	3.355
**Metric**	**Criteria**														
Mean				4.03	9.08	46.87	81.79	529.05	1003.46	-0.30	5200.04	-0.10	9645.09	-0.30	793.78
Std Dev				0.32	1.09	2.68	6.36	46.33	93.18	1.42	354.40	0.74	805.23	0.79	79.90
RSD	< 20%			6.49%				9.27%							
Accuracy	± 30%			80.50%				105.81%							
HR_{PIR}				1.28											
Upper PIR	≤ 150%			106%											
Lower PIR	≥ 50%			55%											

**Assay Name:**	**SRP PFAS**	**New Method Validation**			
**Instrument:**	**Exploris 240**	**Regression:**	**Linear**	**Weighting:**	**$1/x^2$**
**Analyte:**	**PFMOAA**				
**Internal Standard**	**PFBA**				

DAY 1

**Calibrators (ng/L)**	~~1~~	~~2~~	~~5~~	~~10~~	~~50~~	~~100~~	**500**	**1,000**	~~5,000~~	~~10,000~~		

Date 10/27/2021

R^2 0.8619 1

**MRL =**	**500**	**ng/L**	**IDP =**	**6.02%**	**@ 5000 ng/L**
**DL =**	**138.66**	**ng/L**	**IDA =**	**87.60%**	**@ 5000 ng/L**

Rep		1	2	5	10	50	100	500	1,000	Neg	5,000	Neg	10,000	Neg	CON 800
1								414.03	705.26	-33.04	4,702.63	-35.13	7,330.63	-6.37	592.73
2								417.85	888.90	-28.69	4,522.40	-38.59	7,471.86	36.01	623.30
3								*585.68	1,283.00	364.55	6,854.73	304.84	12,506.17	199.06	*1059.97
4								*610.8	1,067.81	206.09	5,200.00	20.32	8,915.72	-31.19	975.29
5								488.41	1,236.39	162.10	4,394.93	20.71	8,269.21	17.04	630.62
6								*654.41	1,067.19	92.14	5,340.31	199.05	10,630.01	230.72	*1071.86
7								*780.57	1,291.13	83.20	5,944.47	122.30	10,681.51	191.80	*1031.47
8								457.18	1,184.98	153.83	5,864.21	168.53	8,450.42	16.21	596.26
9								412.59	828.14	7.44	3,992.90	135.84	8,238.35	150.36	592.62
	N=							5	9	9	9	9	9	9	6
	t value							4.604	3.355	3.355	3.355	3.355	3.355	3.355	4.032
**Metric**	**Criteria**														
Mean								438.01	1061.42	111.96	5201.84	99.76	9165.99	89.29	668.47
Std Dev								30.12	199.68	120.13	854.07	109.72	1635.54	96.24	138.03
RSD	< 20%							6.02%							
Accuracy	± 30%							87.60%							
HR_{PIR}								166.40							
Upper PIR	≤ 150%							121%							
Lower PIR	≥ 50%							54%							

**Assay Name:**	**SRP PFAS**	**New Method Validation**			
**Instrument:**	**Explorис 240**	**Regression:**	**Linear**	**Weighting:**	**$1/x^2$**
**Analyte:**	**NaDONA**				
**Internal Standard**	**GenX Decarb.**				

DAY 1

**Calibrators (ng/L)**	~~1~~	**2**	**5**	**10**	**50**	**100**	**500**	**1,000**	**5,000**	~~10,000~~		

Date 10/27/2021

R^2 0.9993 7

**MRL =**	**2**	**ng/L**	**IDP =**	**3.75%**	**@ 500 ng/L**
**DL =**	**0.80**	**ng/L**	**IDA =**	**105.52%**	**@ 500 ng/L**

Rep		**1**	**2**	**5**	**10**	**50**	**100**	**500**	**1,000**	**Neg**	**5,000**	**Neg**	**10,000**	**Neg**	**CON 800**
1			1.77	5.15	9.71	55.25	107.45	514.76	1,049.65	0.61	5,859.52	0.93	9,693.17	2.13	737.46
2			*2.46	4.82	10.49	55.22	94.88	527.17	1,032.24	0.35	5,622.14	2.10	8,567.30	2.20	736.68
3			*1.47	4.51	9.21	49.90	97.36	518.79	926.42	0.62	5,215.98	1.26	9,994.82	1.81	717.09
4			*2.33	5.57	10.27	54.38	93.00	549.47	938.57	1.27	5,537.85	0.89	8,820.55	1.90	726.38
5			1.82	5.16	8.94	54.01	107.77	512.65	942.09	6.01	5,045.64	0.91	9,661.68	1.70	728.44
6			1.98	5.78	9.64	52.38	103.81	546.74	918.04	0.40	5,052.16	1.10	8,938.10	1.60	731.32
7			2.22	4.86	10.04	51.94	107.24	561.24	928.61	0.36	5,212.85	0.80	9,928.51	1.41	746.73
8			2.26	5.38	10.23	51.18	117.87	511.88	959.62	1.19	5,209.03	1.33	9,525.90	1.66	725.03
9			1.8	5.02	9.52	53.39	112.87	505.80	954.14	0.33	5,358.15	0.82	9,192.88	2.52	728.36
	N=		6	9	9	9	9	9	9	9	9	9	9	9	9
	t value		4.032	3.355	3.355	3.355	3.355	3.355	3.355	3.355	3.355	3.355	3.355	3.355	3.355
**Metric**	**Criteria**														
Mean			1.98	5.14	9.78	53.07	104.69	527.61	961.04	1.24	5345.92	1.13	9369.21	1.88	730.83
Std Dev			0.20	0.37	0.49	1.75	7.83	18.75	44.64	1.72	259.67	0.39	480.77	0.33	8.06
RSD	< 20%		9.96%					3.75%							
Accuracy	± 30%		98.75%					105.52%							
HR_{PIR}			0.94												
Upper PIR	≤ 150%		146%												
Lower PIR	≥ 50%		52%												

**Assay Name:**	**SRP PFAS**	**New Method Validation**			
**Instrument:**	**Exploris 240**	**Regression:**	**Linear**	**Weighting:**	**$1/x^2$**
**Analyte:**	**PEPA**				
**Internal Standard**	**GenX Decarb.**				

DAY 1

**Calibrators (ng/L)**	~~**1**~~	~~**2**~~	~~**5**~~	~~**10**~~	**50**	**100**	**500**	**1,000**	**5,000**	**10,000**		
**Date**	**10/27/2021**											
**R^2**	**0.9998**	**1**										

**MRL =**	**50**	**ng/L**	**IDP =**	**2.49%**	**@ 500 ng/L**
**DL =**	**7.48**	**ng/L**	**IDA =**	**105.02%**	**@ 500 ng/L**

Rep		**1**	**2**	**5**	**10**	**50**	**100**	**500**	**1,000**	**Neg**	**5,000**	**Neg**	**10,000**	**Neg**	**CON 800**
1						54.74	81.62	527.01	1,041.18	4.81	4,777.57	9.55	8,705.79	7.24	789.76
2						52.60	78.47	514.88	980.18	3.97	4,625.25	5.24	9,736.10	6.35	790.75
3						52.71	73.95	543.63	964.09	5.15	4,902.33	3.73	9,257.10	5.35	800.14
4						50.74	85.49	515.53	968.81	6.48	4,607.81	3.78	9,667.92	3.83	773.86
5						52.91	85.26	538.76	971.03	2.05	4,732.44	3.07	9,685.99	4.07	780.73
6						48.25	94.85	512.62	1,021.15	2.78	4,706.51	3.09	10,024.89	4.58	836.96
7						47.71	86.83	542.66	960.12	0.09	4,744.27	1.01	10,197.88	1.77	794.70
8						49.21	99.66	516.80	995.04	0.75	4,754.18	1.26	9,785.18	2.44	790.19
9						50.86	93.91	513.80	985.02	0.25	4,804.21	1.39	9,577.49	2.48	792.40
	N=					9	9	9	9	9	9	9	9	9	9
	t value					3.355	3.355	3.355	3.355	3.355	3.355	3.355	3.355	3.355	3.355
**Metric**	**Criteria**														
Mean						51.08	86.67	525.08	987.40	2.93	4739.40	3.57	9626.48	4.23	794.39
Std Dev						2.23	7.79	12.43	25.92	2.18	84.28	2.49	410.18	1.75	16.70
RSD	< 20%					4.46%		2.49%							
Accuracy	± 30%					102.16%		105.02%							
HR_{PIR}						8.31									
Upper PIR	≤ 150%					119%									
Lower PIR	≥ 50%					86%									

**Assay Name:**	**SRP PFAS**	**New Method Validation**	
**Instrument:**	**Exploris 240**	**Regression:** Linear	**Weighting:** $1/x^2$
**Analyte:**	**PFO3OA**		
**Internal Standard**	**GenX Decarb.**		

DAY 1

Calibrators (ng/L)	~~1~~	~~2~~	~~5~~	~~10~~	50	100	500	1,000	~~5,000~~	~~10,000~~		

Date 10/27/2021

R^2 0.9962 1

**MRL =**	**50**	**ng/L**	**IDP =**	**2.05%**	**@ 500 ng/L**
**DL =**	**12.46**	**ng/L**	**IDA =**	**106.30%**	**@ 500 ng/L**

Rep		1	2	5	10	50	100	500	1,000	Neg	5,000	Neg	10,000	Neg	CON 800
1						52.84	79.13	533.27	1,016.81	3.58	4,603.72	6.81	9,044.78	4.80	818.88
2						57.89	76.30	515.68	1,033.44	3.58	4,906.17	3.58	9,469.55	3.58	802.98
3						53.19	86.41	521.52	1,034.16	5.03	4,920.32	3.58	9,695.00	3.58	803.32
4						47.89	100.01	543.46	1,028.68	7.08	4,768.01	3.58	9,715.65	3.58	796.90
5						53.54	95.14	541.01	1,009.10	5.46	4,887.82	3.58	10,118.58	3.58	801.65
6						48.82	106.51	546.25	1,000.27	3.58	4,922.83	3.58	9,972.58	3.58	828.54
7						54.28	101.25	528.89	955.82	3.58	4,737.98	3.58	9,734.07	3.58	802.04
8						59.90	100.96	533.51	1,017.10	3.58	4,776.66	3.58	9,575.57	4.59	780.31
9						50.31	85.55	519.89	966.12	3.58	4,776.44	3.58	9,257.68	3.58	754.93
	N=					9	9	9	9	9	9	9	9	9	9
	t value					3.355	3.355	3.355	3.355	3.355	3.355	3.355	3.355	3.355	3.355
**Metric**	**Criteria**														
Mean						53.18	92.36	531.50	1006.83	4.34	4811.11	3.94	9620.38	3.83	798.84
Std Dev						3.71	10.19	10.27	26.76	1.19	101.07	1.02	314.01	0.47	20.04
RSD	< 20%					7.42%		2.05%							
Accuracy	± 30%					106.37%		106.30%							
HR_{PIR}						13.84									
Upper PIR	≤ 150%					134%									
Lower PIR	≥ 50%					79%									

**Assay Name:**	**SRP PFAS**	**New Method Validation**	
**Instrument:**	**Exploris 240**	**Regression:** **Linear**	**Weighting:** **$1/x^2$**
**Analyte:**	**PFO4DA**		
**Internal Standard**	**GenX Decarb.**		

DAY 1

**Calibrators (ng/L)**	**~~1~~**	**~~2~~**	**~~5~~**	**10**	**50**	**100**	**500**	**1,000**	**5,000**	**~~10,000~~**		

Date **10/27/2021**

R^2 **0.993** **9**

**MRL =**	**50**	**ng/L**	**IDP =** **10.10%**	**@ 500 ng/L**
**DL =**	**20.34**	**ng/L**	**IDA =** **91.08%**	**@ 500 ng/L**

Rep		**1**	**2**	**5**	**10**	**50**	**100**	**500**	**1,000**	**Neg**	**5,000**	**Neg**	**10,000**	**Neg**	**CON 800**
1						45.92	86.22	406.49	795.92	29.35	4,600.71	13.72	12,429.58	13.72	625.82
2						51.35	71.09	401.66	754.61	13.72	6,086.69	23.74	12,079.08	13.72	638.11
3						59.34	75.89	392.09	773.48	13.72	4,508.07	13.72	12,608.01	13.72	630.88
4						46.23	73.20	513.11	1,128.29	13.72	6,290.55	13.72	12,400.99	13.72	821.98
5						*38.95	100.21	517.17	1,091.49	13.72	4,707.21	16.17	12,082.75	13.72	683.39
6						*39.86	98.84	448.31	1,009.38	13.72	4,544.77	13.72	11,898.17	13.72	710.40
7						52.53	93.72	460.68	833.31	13.72	5,044.40	13.72	13,007.70	13.72	841.04
8						59.75	87.20	530.87	1,035.40	13.72	5,068.44	13.72	11,837.31	13.72	857.28
9						46.86	88.36	428.42	785.29	13.72	4,608.14	13.72	10,669.08	13.72	669.18
	N=					7	9	9	9	9	9	9	9	9	9
	t value					3.707	3.355	3.355	3.355	3.355	3.355	3.355	3.355	3.355	3.355
**Metric**	**Criteria**														
Mean						51.71	86.08	455.42	911.91	15.46	5051.00	15.11	12112.52	13.72	719.79
Std Dev						5.49	10.12	50.48	142.75	4.91	639.24	3.15	618.04	0.00	89.09
RSD	< 20%					10.97%		10.10%							
Accuracy	± 30%					103.42%		91.08%							
HR_{PIR}						23.25									
Upper PIR	≤ 150%					150%									
Lower PIR	≥ 50%					57%									

**Assay Name:**	**SRP PFAS**	**New Method Validation**				
**Instrument:**	**Exploris 240**	**Regression:**	**Linear**	**Weighting:**	**$1/x^2$**	
**Analyte:**	**PFO5DoA**					
**Internal Standard**	**GenX Decarb.**					

DAY 1

**Calibrators (ng/L)**	**~~1~~**	**~~2~~**	**~~5~~**	**~~10~~**	**~~50~~**	**100**	**500**	**1,000**	**5,000**	**~~10,000~~**		

Date **10/27/2021**

R^2 **0.9982** **1**

**MRL =**	**100**	**ng/L**	**IDP =**	**7.58%**	**@ 500 ng/L**
**DL =**	**40.72**	**ng/L**	**IDA =**	**111.36%**	**@ 500 ng/L**

Rep		**1**	**2**	**5**	**10**	**50**	**100**	**500**	**1,000**	**Neg**	**5,000**	**Neg**	**10,000**	**Neg**	**CON 800**
1							86.16	613.85	966.62	(0.35)	4,838.72	130.01	8,414.85	(6.54)	844.31
2							80.11	555.64	1,033.57	(4.56)	4,877.78	(7.78)	10,059.14	(8.49)	873.51
3							99.34	555.45	786.48	(2.79)	3,532.72	(5.98)	8,150.06	(0.87)	673.27
4							92.20	581.41	1,101.94	(0.74)	5,157.27	3.55	9,456.26	(12.93)	828.53
5							87.90	471.24	1,093.26	(12.93)	4,880.13	(3.58)	10,168.68	(1.46)	902.79
6							119.95	591.79	1,057.02	(8.25)	4,992.54	(0.59)	8,666.09	0.90	745.74
7							104.83	537.33	835.82	(5.76)	5,008.41	(6.17)	9,236.17	0.88	725.72
8							90.54	545.29	1,047.50	(12.93)	4,654.21	2.69	9,028.97	(8.44)	875.91
9							110.49	559.32	1,027.86	(12.93)	4,979.58	(8.98)	9,679.45	5.54	827.99
	N=						9	9	9	9	9	9	9	9	9
	t value						3.355	3.355	3.355	3.355	3.355	3.355	3.355	3.355	3.355
**Metric**	**Criteria**														
Mean							96.84	556.81	994.45	-6.80	4769.04	11.46	9206.63	-3.49	810.86
Std Dev							12.14	37.90	105.35	4.89	456.19	42.12	667.17	5.57	73.60
RSD	< 20%						12.14%	7.58%							
Accuracy	± 30%						96.84%	111.36%							
HR_{PIR}							45.25								
Upper PIR	≤ 150%						142%								
Lower PIR	≥ 50%						52%								

Assay Name:	SRP PFAS	New Method Validation			
Instrument:	Exploris 240	Regression:	Linear	Weighting:	$1/x^2$
Analyte:	FHpPA				
Internal Standard	PFUdA				

DAY 1

Calibrators (ng/L)	~~1~~	~~2~~	~~5~~	~~10~~	50	100	500	1,000	5,000	~~10,000~~		

Date 10/27/2021

R^2 0.9948 1

MRL =	50	ng/L	IDP =	8.73%	@ 500 ng/L
DL =	11.93	ng/L	IDA =	100.40%	@ 500 ng/L

															CON
Rep		1	2	5	10	50	100	500	1,000	Neg	5,000	Neg	10,000	Neg	800
1						50.7	76.24	432.22	851.79	26.76	3,660.60	19.10		(12.68)	736.84
2						45.56	57.4	531.01	1,023.91	18.48	3,409.43	15.46		9.53	698.22
3						57.11	68.96	520.35	842.29	4.39	2,686.40	(4.17)		6.71	565.52
4						53.9	70.52	458.26	889.10	19.12	3,975.63	(4.52)		16.63	691.75
5						55.03	89.76	519.58	935.85	32.69	3,953.41	3.40		12.75	772.11
6						56.84	72.95	482.58	954.25	3.30	4,477.31	17.00		(1.52)	548.42
7						52.52	83.54	462.44	858.18	33.04	4,578.47	2.15		19.40	683.43
8						56.93	105.43	579.99	1,078.08	21.64	4,090.17	(6.29)		30.31	843.41
9						51.57	107.57	531.49	1,051.54	(1.40)	4,364.99	8.73		0.57	814.62
	N=					9	9	9	9	9	9	9		9	9
	t value					3.355	3.355	3.355	3.355	3.355	3.355	3.355		3.355	3.355
**Metric**	**Criteria**														
Mean						53.35	81.37	501.99	942.78	17.56	3910.71	5.65		9.08	706.04
Std Dev						3.56	15.93	43.67	85.19	12.06	560.19	9.28		11.98	95.07
RSD	< 20%					7.11%		8.73%							
Accuracy	± 30%					106.70%		100.40%							
HR_{PIR}						13.26									
Upper PIR	≤ 150%					133%									
Lower PIR	≥ 50%					80%									

**Assay Name:**	**SRP PFAS**	**New Method Validation**	
**Instrument:**	**Exploris 240**	**Regression:** **Linear**	**Weighting:** **$1/x^2$**
**Analyte:**	**N-AP-FHxSA**		
**Internal Standard**	**PFUdA**		

DAY 1

**Calibrators (ng/L)**	~~1~~	~~2~~	~~5~~	**10**	**50**	**100**	**500**	**1,000**	**5,000**	~~10,000~~		
**Date**	**10/27/2021**											
**R^2**	**0.9987**	1										

**MRL =**	**10**	**ng/L**	**IDP =**	**16.84%**	**@ 500 ng/L**
**DL =**	**2.08**	**ng/L**	**IDA =**	**115.62%**	**@ 500 ng/L**

Rep		**1**	**2**	**5**	**10**	**50**	**100**	**500**	**1,000**	**Neg**	**5,000**	**Neg**	**10,000**	**Neg**	**CON 800**
1					11.73	49.99	66.03	417.31	927.50	2.20	4,558.93	5.70		5.68	715.13
2					12.07	45.44	66.30	564.35	1,008.15	2.73	3,326.17	3.93		7.26	697.16
3					12.47	53.41	70.36	680.35	1,071.77	3.74	5,094.94	4.22		6.81	856.33
4					*14.6	64.45	96.78	639.12	1,004.49	20.71	3,967.78	3.99		9.17	948.55
5					12.10	53.97	81.69	599.14	1,124.37	3.16	3,850.55	3.55		7.45	801.96
6					11.94	49.74	102.01	650.13	1,156.47	2.76	4,589.64	4.51		6.78	800.06
7					13.43	61.94	95.78	639.35	1,072.98	3.06	4,724.14	4.17		7.57	975.30
8					13.26	57.17	120.93	551.18	1,357.69	3.76	5,026.48	5.32		7.27	748.96
9					11.99	54.14	91.67	461.87	976.09	2.16	4,268.99	4.34		6.75	742.09
	N=				8	9	9	9	9	9	9	9		9	9
	t value				3.499	3.355	3.355	3.355	3.355	3.355	3.355	3.355		3.355	3.355
**Metric**	**Criteria**														
Mean					12.37	54.47	87.95	578.09	1077.72	4.92	4378.62	4.41		7.19	809.50
Std Dev					0.59	5.66	17.44	84.22	120.15	5.61	547.38	0.65		0.88	93.63
RSD	< 20%				5.94%			16.84%							
Accuracy	± 30%				123.74%			115.62%							
HR_{PIR}					2.34										
Upper PIR	≤ 150%				147%										
Lower PIR	≥ 50%				100%										

**Assay Name:**	**SRP PFAS**	**New Method Validation**			
**Instrument:**	**Exploris 240**	**Regression:**	**Linear**	**Weighting:**	**$1/x^2$**
**Analyte:**	**N-CMAmP-6:2FOSA (6:2 FTAB)**				
**Internal Standard**	**8:2 FTS**				

DAY 1

**Calibrators (ng/L)**	**~~1~~**	**~~2~~**	**~~5~~**	**10**	**50**	**100**	**500**	**1,000**	**5,000**	**10,000**		

Date **10/27/2021**

R^2 **0.9963** **1**

**MRL =**	**10**	**ng/L**	**IDP =**	**12.49%**	**@ 500 ng/L**
**DL =**	**4.26**	**ng/L**	**IDA =**	**95.69%**	**@ 500 ng/L**

Rep		**1**	**2**	**5**	**10**	**50**	**100**	**500**	**1,000**	**Neg**	**5,000**	**Neg**	**10,000**	**Neg**	**CON 800**
1					9.68	55.64	85.70	548.89	1,064.77	0.79	4,793.95	3.27	9,344.39	2.48	802.28
2					9.12	47.37	79.96	563.57	1,063.56	0.79	5,183.87	0.79	9,068.52	1.26	849.22
3					11.88	44.95	78.82	443.82	810.55	1.79	3,383.35	0.79	6,804.37	0.79	492.67
4					8.82	30.03	57.81	410.45	850.87	5.64	4,688.40	0.79	8,950.81	2.27	625.25
5					11.81	52.11	90.96	421.17	892.07	0.79	5,435.67	0.79	9,717.81	0.79	799.56
6					*6.51	41.71	72.98	431.74	958.91	0.79	5,267.83	1.31	8,398.63	1.44	611.25
7					9.84	42.67	68.86	467.09	889.94	0.79	4,898.13	0.79	6,928.22	1.72	580.44
8					8.49	47.58	98.80	440.79	913.01	1.55	4,096.93	0.79	9,653.94	1.98	867.60
9					10.69	53.93	106.07	578.63	1,102.96	0.79	5,148.77	0.79	9,179.27	1.41	906.65
	N=				8	9	9	9	9	9	9	9	9	9	9
	t value				3.499	3.355	3.355	3.355	3.355	3.355	3.355	3.355	3.355	3.355	3.355
**Metric**	**Criteria**														
Mean					10.04	46.22	82.22	478.46	949.63	1.52	4766.32	1.12	8671.77	1.57	726.10
Std Dev					1.22	7.32	14.18	62.43	98.40	1.50	615.09	0.78	1033.11	0.56	140.65
RSD	< 20%				12.17%			12.49%							
Accuracy	± 30%				100.41%			95.69%							
HR_{PIR}					4.79										
Upper PIR	≤ 150%				148%										
Lower PIR	≥ 50%				53%										

**Assay Name:**	**SRP PFAS**	**New Method Validation**	
**Instrument:**	**Explorис 240**	**Regression:** **Linear**	**Weighting:** $1/x^2$
**Analyte:**	**N-TAmP-FHxSA**		
**Internal Standard**	**PFOSA**		

DAY 1

**Calibrators (ng/L)**	~~1~~	~~2~~	~~5~~	**10**	**50**	**100**	**500**	**1,000**	**5,000**	~~10,000~~		

Date 10/27/2021

R^2 0.9988 3

**MRL =**	**10**	**ng/L**	**IDP =**	**11.86%**	**@ 500 ng/L**
**DL =**	**2.74**	**ng/L**	**IDA =**	**110.83%**	**@ 500 ng/L**

Rep		1	2	5	10	50	100	500	1,000	Neg	5,000	Neg	10,000	Neg	CON 800
1					*14.24	61.83	107.26	644.18	1,232.80	0.99	5,176.84	1.00		1.59	757.52
2					11.02	45.55	78.28	577.17	1,216.18	1.10	5,056.06	4.80		1.23	977.56
3					*14.19	48.65	71.47	490.65	931.44	3.04	5,533.13	1.39		1.84	880.12
4					*13.63	53.25	114.19	542.22	876.50	14.73	4,258.88	0.84		1.40	727.65
5					*12.65	44.61	76.97	506.98	874.19	0.71	5,184.90	1.23		1.42	717.37
6					9.90	44.72	84.36	552.50	1,131.02	0.88	5,144.01	1.05		1.14	745.01
7					11.60	56.98	110.31	508.53	1,061.97	0.90	4,593.29	1.22		1.44	1,062.08
8					11.44	43.73	94.23	502.36	933.62	1.85	4,237.75	1.45		1.48	896.18
9					11.03	63.13	118.33	662.89	1,249.42	1.11	5,637.48	1.08		1.41	1,007.77
	N=				5	9	9	9	9	9	9	9		9	9
	t value				4.604	3.355	3.355	3.355	3.355	3.355	3.355	3.355		3.355	3.355
**Metric**	**Criteria**														
Mean					11.00	51.38	95.04	554.16	1056.35	2.81	4980.26	1.56		1.44	863.47
Std Dev					0.59	7.24	16.90	59.28	147.48	4.27	479.83	1.16		0.19	124.58
RSD	< 20%				5.94%			11.86%							
Accuracy	± 30%				109.98%			110.83%							
HR_{PIR}					3.28										
Upper PIR	≤ 150%				143%										
Lower PIR	≥ 50%				77%										

**Assay Name:**	**SRP PFAS**	**New Method Validation**	
**Instrument:**	**Exploris 240**	**Regression:** **Linear**	**Weighting:** **1/x^2**
**Analyte:**	**PS Acid**		
**Internal Standard**	**PFOS**		

DAY 1

**Calibrators (ng/L)**	~~**1**~~	~~**2**~~	**5**	**10**	**50**	**100**	**500**	**1,000**	**5,000**	**10,000**		

Date 10/27/2021

R^2 0.9906 1

**MRL =**	**5**	**ng/L**	**IDP =**	**2.65%**	**@ 500 ng/L**
**DL =**	**0.70**	**ng/L**	**IDA =**	**114.58%**	**@ 500 ng/L**

Rep		**1**	**2**	**5**	**10**	**50**	**100**	**500**	**1,000**	**Neg**	**5,000**	**Neg**	**10,000**	**Neg**	**CON 800**
1				4.2	8.31	49.08	81.73	560.82	1,018.29	(1.27)	5,012.85	1.07	8,434.74	(0.60)	823.29
2				4.73	8.97	61.40	80.29	574.40	1,067.60	(1.08)	5,187.00	(0.65)	9,105.27	0.39	805.82
3				4.14	8.94	53.06	80.51	553.65	1,127.43	0.74	5,146.08	(0.89)	8,848.74	(0.43)	824.87
4				4.41	9.1	50.61	97.19	569.89	1,026.10	7.49	5,200.82	(0.68)	10,188.86	(0.27)	865.38
5				4.18	8.6	51.58	90.04	578.19	1,082.89	(1.28)	4,974.38	(0.93)	9,864.03	0.19	851.33
6				4.43	10.54	53.25	102.17	565.90	1,076.47	(1.41)	5,576.89	(0.59)	9,209.10	(0.28)	830.01
7				4.16	8.87	54.23	97.61	604.02	1,118.73	(1.41)	5,061.41	(0.63)	9,008.65	(0.52)	871.69
8				4.55	9.74	53.95	103.53	574.67	1,118.24	(0.19)	5,173.77	(0.14)	9,795.76	0.34	871.35
9				4.63	8.23	51.37	100.47	574.54	1,098.77	(0.99)	5,292.48	(1.01)	8,890.97	0.32	834.67
	N=			9	9	9	9	9	9	9	9	9	9	9	9
	t value			3.355	3.355	3.355	3.355	3.355	3.355	3.355	3.355	3.355	3.355	3.355	3.355
**Metric**	**Criteria**														
Mean				4.38	9.03	53.17	92.62	572.90	1081.61	0.07	5180.63	-0.49	9260.68	-0.10	842.05
Std Dev				0.21	0.68	3.31	9.08	13.25	37.16	2.71	168.70	0.60	536.53	0.38	22.43
RSD	< 20%			4.19%				2.65%							
Accuracy	± 30%			87.62%				114.58%							
HR_{PIR}				0.78											
Upper PIR	≤ 150%			103%											
Lower PIR	≥ 50%			72%											

**Assay Name:**	**SRP PFAS**	**New Method Validation**	
**Instrument:**	**Exploris 240**	**Regression:** Linear	**Weighting:** $1/x^2$
**Analyte:**	**Nafion byproduct 2**		
**Internal Standard**	**PFOS**		

DAY 1

**Calibrators (ng/L)**	~~1~~	2	5	10	50	100	500	1,000	5,000	10,000		

Date 10/27/2021

R^2 0.9966 1

**MRL =**	**2**	**ng/L**	**IDP =**	**3.24%**	**@ 500 ng/L**
**DL =**	**0.41**	**ng/L**	**IDA =**	**111.76%**	**@ 500 ng/L**

Rep		1	2	5	10	50	100	500	1,000	Neg	5,000	Neg	10,000	Neg	CON 800
1			2.00	5.92	10.61	44.85	77.00	569.20	933.83	0.66	4,173.77	2.95	10,041.98	1.42	862.47
2			2.23	4.92	9.95	53.26	76.52	584.88	1,106.81	0.78	6,012.92	1.05	10,044.87	1.31	814.60
3			*2.74	4.91	9.51	54.46	80.79	521.81	1,148.02	2.07	5,607.84	1.19	10,955.52	1.94	879.06
4			2.38	4.75	10.65	46.98	96.43	559.91	1,073.84	8.47	6,015.73	1.02	9,020.58	1.13	889.55
5			2.08	5.06	9.63	49.57	91.74	548.81	1,103.08	0.56	5,958.89	0.86	10,347.12	1.81	850.73
6			2.10	5.27	10.56	51.03	102.65	561.58	1,057.48	0.78	6,254.01	1.24	10,487.95	1.70	795.84
7			2.21	5.92	10.52	55.43	93.19	556.85	1,167.19	0.56	5,985.58	0.94	10,335.97	1.78	894.07
8			2.30	5.11	10.24	53.57	96.17	568.63	1,131.53	0.84	6,095.64	1.50	10,932.45	1.19	880.02
9			2.15	5.88	11.13	50.05	96.22	557.44	1,111.16	0.56	6,176.22	0.88	10,493.62	2.10	833.90
	N=		8	9	9	9	9	9	9	9	9	9	9	9	9
	t value		3.499	3.355	3.355	3.355	3.355	3.355	3.355	3.355	3.355	3.355	3.355	3.355	3.355
**Metric**	**Criteria**														
Mean			2.18	5.30	10.31	51.02	90.08	558.79	1092.55	1.70	5808.96	1.29	10295.56	1.60	855.58
Std Dev			0.12	0.45	0.50	3.34	8.99	16.22	64.64	2.44	602.54	0.62	545.12	0.33	32.61
RSD	< 20%		5.80%					3.24%							
Accuracy	± 30%		109.06%					111.76%							
HR_{PIR}			0.46												
Upper PIR	≤ 150%		132%												
Lower PIR	≥ 50%		86%												

**Assay Name:**	**SRP PFAS**	**New Method Validation**					
**Instrument:**	**Exploris 240**	**Regression:**	**Linear**	**Weighting:**	**$1/x^2$**		
**Analyte:**	**F53B Minor (11Cl-PF3OUdS)**						
**Internal Standard**	**10:2 FTS**						

DAY 1

**Calibrators (ng/L)**	~~**1**~~	**2**	**5**	**10**	**50**	**100**	**500**	**1,000**	**5,000**	~~**10,000**~~		

Date **10/27/2021**

R^2 **1** **1**

**MRL =**	**2**	**ng/L**	**IDP =**	**2.96%**	**@ 500 ng/L**
**DL =**	**0.71**	**ng/L**	**IDA =**	**105.55%**	**@ 500 ng/L**

Rep		**1**	**2**	**5**	**10**	**50**	**100**	**500**	**1,000**	**Neg**	**5,000**	**Neg**	**10,000**	**Neg**	**CON 800**
1			1.82	4.65	9.72	53.39	80.62	519.79	1,034.22	0.85	4,965.97	8.38		4.60	814.95
2			1.85	5.3	11.40	58.53	77.55	528.58	1,067.49	0.67	4,882.18	3.50		5.89	835.40
3			1.94	4.65	10.89	54.40	76.84	525.41	1,033.86	3.32	5,009.52	3.50		6.39	816.99
4			2.05	4.63	9.68	52.88	91.27	506.60	1,081.76	5.13	5,277.11	2.61		4.82	777.02
5			2.36	4.78	9.65	51.88	84.21	512.77	937.13	0.76	5,074.76	3.35		5.42	809.23
6			1.72	4.45	10.90	52.33	101.56	547.42	1,052.06	0.78	4,807.28	3.26		4.68	834.63
7			2.11	4.34	11.61	53.54	90.60	555.15	1,019.60	0.82	4,923.55	3.92		4.42	794.11
8			1.95	4.98	10.03	53.02	100.26	533.85	1,030.11	2.16	5,034.65	4.16		5.76	800.13
9			2.35	4.87	10.46	53.55	94.81	520.07	987.05	0.84	5,086.67	3.17		6.05	790.14
	N=		9	9	9	9	9	9	9	9	9	9		9	9
	t value		3.355	3.355	3.355	3.355	3.355	3.355	3.355	3.355	3.355	3.355		3.355	3.355
**Metric**	**Criteria**														
Mean			2.02	4.74	10.48	53.72	88.64	527.74	1027.03	1.70	5006.85	3.98		5.34	808.07
Std Dev			0.21	0.27	0.71	1.83	8.80	14.81	40.96	1.48	128.58	1.61		0.68	18.63
RSD	< 20%		10.59%					2.96%							
Accuracy	± 30%		100.83%					105.55%							
HR_{PIR}			0.79												
Upper PIR	≤ 150%		140%												
Lower PIR	≥ 50%		61%												

**Assay Name:**	**SRP PFAS**	**New Method Validation**			
**Instrument:**	**Exploris 240**	**Regression:**	**Linear**	**Weighting:**	**$1/x^2$**
**Analyte:**	**F53B Major (9Cl-PF3ONS)**				
**Internal Standard**	**8:2 FTS**				

DAY 1

**Calibrators (ng/L)**	~~**1**~~	**2**	**5**	**10**	**50**	**100**	**500**	**1,000**	**5,000**	**10,000**		

Date 10/27/2021

R^2 0.9826 1

**MRL =**	**2**	**ng/L**	**IDP =**	**8.81%**	**@ 500 ng/L**
**DL =**	**0.71**	**ng/L**	**IDA =**	**93.71%**	**@ 500 ng/L**

Rep		**1**	**2**	**5**	**10**	**50**	**100**	**500**	**1,000**	**Neg**	**5,000**	**Neg**	**10,000**	**Neg**	**CON 800**
1			1.75	4.66	12.07	52.69	79.33	*661.81	957.58	0.65	4,518.28	7.84	10,877.31	4.62	937.74
2			2.17	5.07	10.47	63.61	80.34	*656.45	1,192.04	0.51	6,001.27	2.86	8,784.68	4.41	1,000.10
3			1.83	4.74	9.02	57.27	95.47	422.38	943.19	2.45	3,899.26	2.09	8,321.21	3.31	747.98
4			1.94	3.86	10.56	38.35	84.98	533.84	1,109.90	10.85	5,543.75	2.42	8,571.17	3.66	750.34
5			1.78	4.97	9.75	50.40	105.87	413.77	1,034.88	0.39	6,123.77	2.80	11,181.66	3.70	974.34
6			*1.46	4.68	9.34	54.35	90.08	491.56	920.32	0.52	6,008.87	2.03	8,831.22	3.35	601.86
7			*2.5	5.41	8.29	50.17	87.03	446.26	1,045.46	0.43	5,772.94	2.07	7,499.68	2.68	773.79
8			1.60	3.76	8.89	55.98	96.36	503.57	1,035.99	1.43	4,761.52	2.41	9,434.11	5.31	1,002.62
9			*2.56	5.71	12.11	50.26	120.76	*657.63	1,249.05	0.30	6,219.81	2.41	10,510.29	3.99	999.46
	N=		6	9	9	9	9	6	9	9	9	9	9	9	9
	t value		4.032	3.355	3.355	3.355	3.355	4.032	3.355	3.355	3.355	3.355	3.355	3.355	3.355
**Metric**	**Criteria**														
Mean			1.85	4.76	10.06	52.56	93.36	468.56	1054.27	1.95	5427.72	2.99	9334.59	3.89	865.36
Std Dev			0.18	0.60	1.28	6.47	12.51	44.06	105.64	3.21	783.04	1.74	1188.66	0.75	140.21
RSD	< 20%		8.85%					8.81%							
Accuracy	± 30%		92.25%					93.71%							
HR_{PIR}			0.83												
Upper PIR	≤ 150%		134%												
Lower PIR	≥ 50%		51%												
